# Comparing the molecular correlates of PTSD in first responders and the broader molecular PTSD literature: a hybrid review

**DOI:** 10.3389/fpsyg.2026.1875411

**Published:** 2026-08-20

**Authors:** Christina E. Grillakis, Stephanie V. Madden, Leslie A. Brick, Sarah M. Merrill

**Affiliations:** 1 Department of Psychology, University of Massachusetts Lowell, Lowell, MA, United States; 2 Department of Psychology, University of New Mexico, Albuquerque, NM, United States

**Keywords:** biological age, DNA methylation, first responders, PTSD, RNA transcription

## Abstract

First responders are trained civilian professionals, including firefighters, law enforcement, emergency medical services, and frontline medical workers, who have a vocational duty and are expected to respond in person to emergency situations. The chronic exposure to adverse traumatic events while working with public health and safety can lead to the clinical presentation of post-traumatic stress disorder (PTSD), which also occurs in other trauma-exposed populations such as military personnel, veterans, refugees, and individuals who experience interpersonal violence. Examining epigenetic and transcriptomic profiles through DNA methylation (DNAm) and RNA transcripts allows researchers to observe how genetic predispositions interact with environmental exposures, shaping biological processes that underlie PTSD across time frames. This review synthesized published analyses on first responders to assess if the observed epigenetic and transcriptomic signatures of PTSD were shared with or distinct from the potential convergence in evidence within the broader molecular PTSD literature as derived from reviews and meta-analyses. In comparing molecular profiles of PTSD across populations, this review assessed the value of integrating epigenetic and transcriptomic markers alongside clinical assessments to understand molecular adaptations, protective and risk factors, and resilience. With the exception of consistently privileged candidate genes, such as *NR3C1* and *FKBP5*, individual studies identified unique DNA methylation sites and transcripts, likely reflecting differences in statistical significance and replication. This highlights the need for standardized methodologies across trauma-exposed populations. Future research should address chronic occupational trauma in first responders to better characterize PTSD manifestation in this unique population and utilize molecular profiling to identify distinct epigenetic and transcriptomic signatures associated with symptom development, treatment trajectories, and risk of reemergence. The field has the potential to inform our understanding of the long-term health consequences and treatment of PTSD for first responders and all those exposed to trauma.

## Graphical abstract

**Figure:**
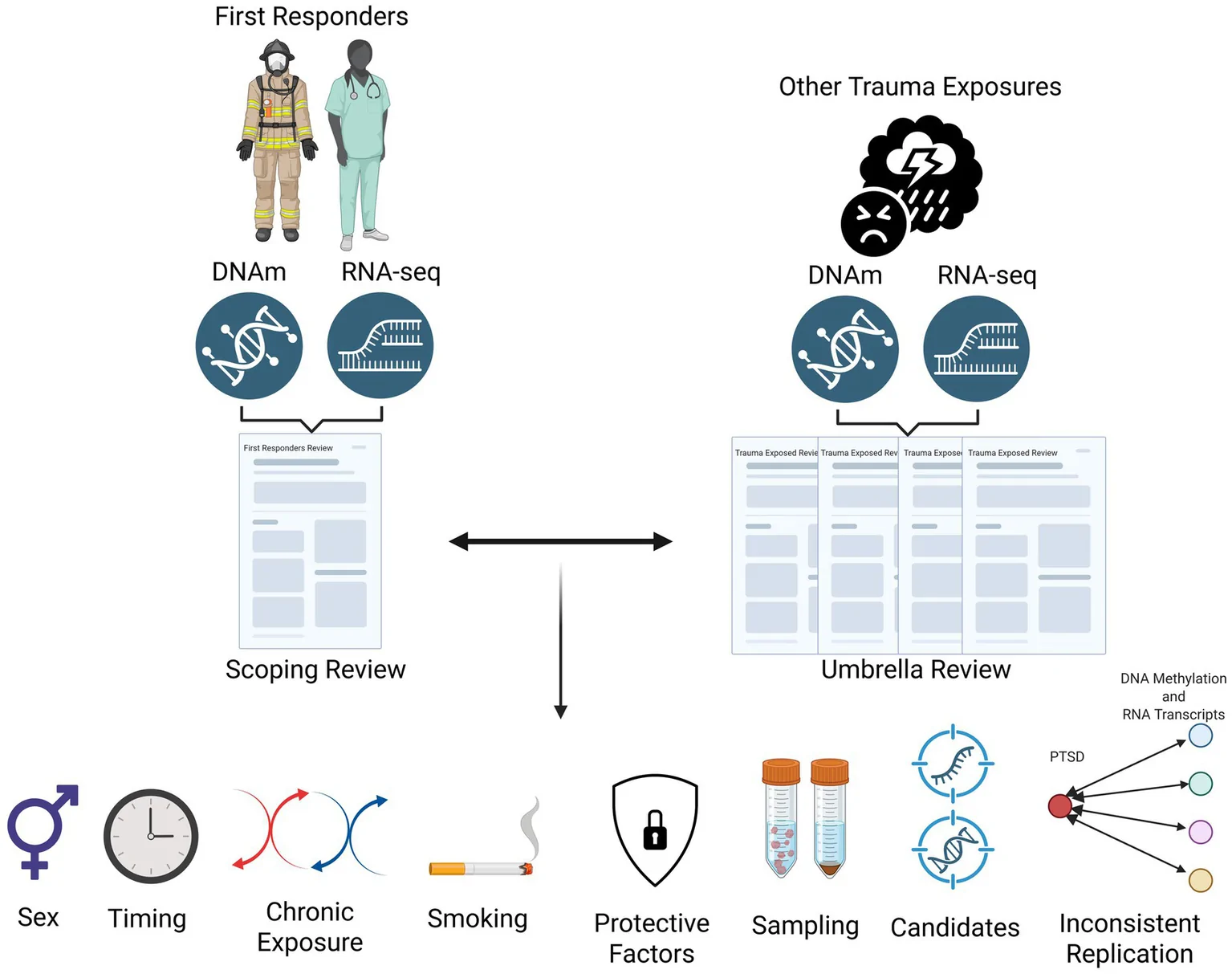
Graphical abstract of the current reviews. First, we conducted a scoping review on DNAm and RNA transcript associations with PTSD within the first responder population. We looked at all original research that met our criteria. Then we conducted an umbrella review, which is a review of reviews and meta-analyses, on all other types of trauma-exposed populations, which were almost always combined. We compared those to outline the key similarities and differences in the available literature, which are visually represented with icons.

## 1 Introduction

First responders are at the forefront of public health and safety and make constant sacrifices by putting the well-being of others before their own. First responders are individuals in professions such as law enforcement, firefighting, emergency medical services, and frontline medical work who are expected to endure critical incidents as part of their occupation, including, but not limited to, vehicle collisions, opioid overdoses, terror attacks, and suicides (Tofighi et al., 2021; Nelson et al., 2020; Nicholson et al., 2023; Skogstad et al., 2015). Cross-nationally, the lifetime prevalence of PTSD in the general population is 3.9% (Koenen et al., 2017); however, within the first responder population, the lifetime prevalence of PTSD is 13.6% (Arena et al., 2025). One possible explanation for this difference is the cumulative nature of occupation-specific trauma experienced by first responders (Lewis-Schroeder et al., 2018), in which even their routine exposure to “low-threshold traumatic events” is a risk factor towards the development of PTSD (Hoell et al., 2023). Furthermore, this amassed exposure leads to a majority of first responders failing to fully return to work following a traumatic event, even after rehabilitation (Gross et al., 2021). The higher prevalence and distinct characteristics of PTSD in first responders highlight a difference from the general population and the need for a culturally sensitive approach when conducting research within the first responder community.

In addition to prevalence, prior research suggests that first responders may present with distinct clinical profiles (Bryant, 2022). The symptoms of PTSD are grouped into four main clusters, including intrusion (Criteria B), avoidance (Criteria C), negative changes in cognitions and mood (Criteria D), and arousal and reactivity (Criteria E) (American Psychiatric Association, 2022). One study found that, compared to civilians with PTSD, first responders were more likely to present with dysphoric symptoms within their diagnosis, including diminished interest, emotional numbing, social detachment, and less psychological reactivity and avoidance of situations (Bryant, 2022). Research illustrates that the stigma and barriers individuals in this profession face are associated with lower rates of mental health care and thus, higher rates of chronic psychopathology (Haugen et al., 2017). Many first responders refrain from seeking help and reporting symptoms due to the fear of potential professional repercussions, such as being seen as unfit for duty or failing to meet requirements to carry a firearm, which is necessary for some occupations (Lewis-Schroeder et al., 2018). Additionally, individuals in this profession face unique vocational challenges, such as dehumanization, due to the general population holding expectations of perfection during times of high stress and trauma respondence, often noted as the superhero stereotype (Mika-Lude et al., 2023). With first responders being leaders in public health and safety, the communities they serve benefit from their mental well-being and thus are ethically and financially responsible for establishing a basis of understanding towards their unique manifestation of PTSD.

In recent literature, moral injury and moral distress have been increasingly recognized as prominent psychological consequences of first responders’ repeated exposure to ethically challenging and traumatic incidents (Barnes et al., 2019; Rimon and Shelef, 2025; Papazoglou and Chopko, 2017; Papazoglou et al., 2020). Although moral injury and moral distress appear to be similar, they are conceptually distinct (Papazoglou and Chopko, 2017). Rimon and Shelef (2025) defines moral injury as “profound psychological distress that arises from perceived violations of one’s ethical or moral code in complex, high-stakes situations”. On the other hand, while the definition of moral distress has evolved over time, it can be described as psychological discomfort experienced by caregiving professionals when they are unable to act according to their ethical and moral code due to factors such as institutional policy, errors in judgement, or circumstances beyond their control (Papazoglou and Chopko, 2017). Moral distress and moral injury could potentially be intertwined and lead to the experience of compassion fatigue, and together, these may serve as risk factors for PTSD susceptibility and severity (Papazoglou and Chopko, 2017). While the early definitions of moral injury originated from studies on veterans (Barnes et al., 2019), it is often applied to research on first responders as well. This supports the notion that there is overlap between military and first responder occupational trauma and PTSD; however, there are distinctions that lead to the separation of these populations in some PTSD research. While both populations face occupational stressors (Walker et al., 2016), military personnel often face deployment-related experiences such as firefights, explosions, exposure to serious death, exposure to toxic substances, or witnessing human suffering (Held et al., 2018). This differs from the predominantly civilian-based exposures that first responders experience, which is why in this review, military populations are included in the broader molecular PTSD literature umbrella review.

The unique presentation of PTSD observed among first responders could be partially explained by the gene–environment correlation (rGE) and gene–environment interaction (GxE). The well-established concept of rGE proposes that genetic differences contribute to the environments individuals self-select into or are exposed to (Jaffee and Price, 2007). For instance, individuals with genetic predispositions associated with traits such as resilience and risk-taking may be more likely to self-select into high-risk occupations, such as emergency response work. From a GxE perspective, first responders’ genetic differences have the potential to influence their response to traumatic events, thus impacting susceptibility to disorders such as PTSD (Jaffee and Price, 2007). Together, these illustrate the dynamic interaction between environmental experiences and genetic factors.

Epigenetic and transcriptomic mechanisms are part of this molecular landscape and are known to reflect the influences of environmental experience on gene expression (Duncan et al., 2014; Dong and Chen, 2013). Epigenetics is the study of chemical modifications of DNA, noting that the DNA sequence itself is not altered (Farsetti et al., 2023). Transcriptomics focuses on gene expression at the RNA level, studying the complete set of RNA transcripts in a specific cell type or tissue under specific physiological conditions (Dong and Chen, 2013). In humans, a primary focus is DNA methylation (DNAm), one epigenetic mechanism that can interact with the binding of transcription factors, potentially impacting transcriptional activity (Comendul et al., 2025). Although epigenetic and transcriptomic factors are both common pathways through which environments and experiences, like trauma, can become biologically embedded (Aristizabal et al., 2020), or get under the skin (Hertzman and Boyce, 2010), they often operate on different molecular time scales (Liu et al., 2024; Trerotola et al., 2015).

DNAm, sometimes referred to as cellular “memory” (Raynal et al., 2012), is a stable and mitotically heritable (Trerotola et al., 2015) biomarker that can reflect enduring and persistent biological changes associated with prior environmental exposures (Nwanaji-Enwerem and Colicino, 2020; Ladd-Acosta and Fallin, 2019). Because of this, DNAm may be helpful in identifying molecular correlates of antecedent risk, such as with trauma history and early life adversity, or biological susceptibility to stress-related psychopathology (Cecil et al., 2020; Zannas and West, 2014). This perspective is in line with the Developmental Origins of Health and Disease (DOHaD) hypothesis, which posits that early life environmental exposures during critical developmental periods can induce biological adaptations that could potentially alter susceptibility to diseases later in life (Lacagnina, 2020). In contrast, transcriptomic profiles provide a “snapshot in time” and, through technologies like RNA-seq, can reveal gene activity at a particular time point across different tissues and conditions (Lowe et al., 2017). Examining stable DNAm patterns alongside dynamic transcriptional activity provides a more comprehensive view of an individual’s molecular landscape by integrating enduring biological risk with active regulatory processes.

This calls for a focus on first responders as an ideal group to examine the impact of repeated adult exposure to trauma on molecular biology. In a profession that demands the constant response to traumatic events, understanding the etiology and developmental pathology of PTSD at the molecular level will allow researchers to better understand the population-level effects of occupational trauma exposure. This could potentially further our understanding of first responders’ heightened risk, or conveyed resilience, in developing the disorder and subsequently ensuring their ability to acquire resources to combat this risk. Examining epigenetic changes and transcriptomic profiles, through DNAm and RNA transcripts, can allow researchers to observe how trauma can interact with genetic predispositions and one’s environment to shape biological factors that underlie PTSD across time frames and in response to treatment.

A systematic review published recently compiled studies that focus on gene expression and epigenetic changes in first responders in relation to PTSD, depression, and anxiety. Of the findings focusing on PTSD, candidate genes were listed as well as vulnerabilities and common themes seen in this population (Alahmad et al., 2025). Our current review extended their work with a focus on only PTSD, and aimed to contextualize the extent to which first responder literature aligns with a synthesis of the broader molecular associations of PTSD literature. This was conducted through a hybrid review design, combining a scoping review of original studies on first responders with an umbrella review, a review of reviews (Aromataris et al., 2020), of meta-analyses and reviews available on the broader molecular PTSD literature. DNAm and RNA transcripts are the primary focus of this review; however, because these molecular processes do not necessarily reflect biological functioning directly, associations with other biomarkers and biological pathways are included to provide additional biological context. There is currently no existing literature that examines epigenetic and transcriptomic signatures of PTSD across populations in relation to first responders. For the purpose of this review, the broader molecular PTSD literature included analyses of individuals who have experienced trauma but did not meet our definition of a first responder. This comparison uses first responders as an exemplar population to examine whether PTSD-associated molecular signatures generalized across trauma-exposed individuals or were dependent upon population-specific features of trauma exposure, including timing, chronicity, moral injury, comorbidities, and the prevalence of and receptivity to treatment.

## 2 Methodology

A preliminary search for existing reviews on this topic was conducted through searching CINAHL, PubMed, PsycInfo, and PTSDpubs and yielded no results. This review followed the protocol in compliance with the JBI Manual for Evidence Synthesis (Aromataris et al., 2020). To represent the screening phase and the selection process, the PRISMA Flow Diagram (Haddaway et al., 2022) was utilized. The assessment of methodological quality was conducted through both the JBI Checklist for Analytical Cross Sectional Studies for original studies (Barker et al., 2026) and the JBI Checklist for Systematic Reviews for reviews and meta-analyses (Aromataris et al., 2015). The review was preregistered through OSF.

### 2.1 First responder scoping review selection and search strategy

The initial scoping review was conducted using existing original research on biological markers associated with first responders who met a clinically relevant threshold for PTSD. For the purpose of this review, “first responders” were: “Trained civilian professionals, including firefighters, law enforcement, emergency medical services, and frontline medical workers who have a vocational duty and are expected to respond in person to emergency situations.” Additionally, the biomarkers assessed included DNAm, RNA transcription, and biological age as derived from DNA methylation and RNA transcripts.

The criteria used to select first responder studies for inclusion in the review were as follows:

- Only original research was included
- Only papers published after 2012 were included
- Only human studies were included
- Only subjects meeting the definition of first responders were included, omitting individuals in combat positions
- The study population must consist of individuals who meet a clinically relevant threshold of PTSD, established using standard diagnostic criteria
- The study must measure one or more biomarkers that are included in a model with PTSD
- Can include both genetic and epigenetic research, if the data is separated and conclusions are separated
- Both biological marker and PTSD diagnostic criteria must be present for the same individual.

The PubMed, CINAHL, PTSDpubs, and APA PsycInfo literature databases were searched using the key words (“first responders” OR “paramedics” OR “firefighters” OR “police officers” OR “emergency medical technician” OR “nurses” OR “paramedicine” OR “World Trade Center Responders” OR “law enforcement” OR “rescue” OR “nursing” OR “emergency” OR “disaster” OR “search and rescue”) AND (“post-traumatic stress disorder” OR “PTSD” OR “posttraumatic stress disorder” OR “post traumatic stress disorder” OR “post traumatic stress symptoms” OR “PTSS” OR “post-traumatic stress symptoms” OR “acute stress disorder”) AND (“epigenetic*” OR “transcriptom*” OR “mRNA” OR “DNAm” OR “DMAme” OR “transcriptomic clock” OR “EAA” OR “epigenetic age” OR “DNA methylation clock” OR “DNA methylation age” OR “transcriptomic age” OR “DNA methylation” OR “biological embedding” OR “molecular embedding” OR “gene expression” OR “RNA expression” OR “epigenetic clock” OR “GrimAge” OR “PhenoAge” OR “FitAge” OR “Horvath” OR “DunedinPACE” OR “Pace of Aging” OR “biological age”).

**Figure 1:**
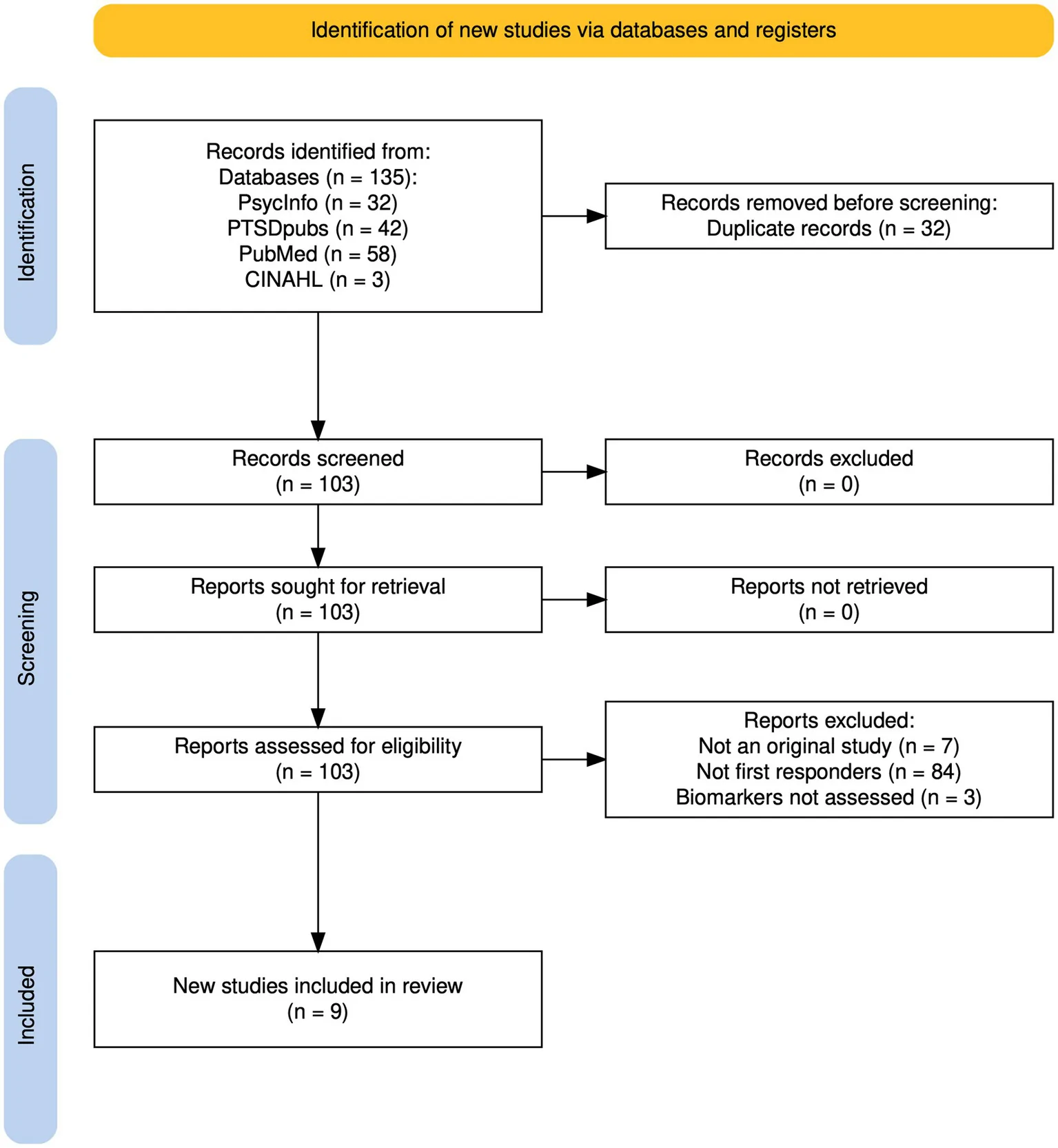
Flow diagram of the selection process of the nine selected studies for the scoping review. Each box indicates a step in the flow diagram of the selection process. The right side indicates how many studies were excluded in each step, while the left shows how many papers remained. A total of 135 records were identified, and 32 duplicates were removed before screening. 103 reports were assessed for eligibility, but 94 were then excluded, leading to nine studies being included in the scoping review for the first responder population. *During the title and abstract screening phase, no studies were excluded. Authors CEG and SVM reviewed the full texts due to the difficulty of screening from title and abstract alone and due to the limited number of papers available.

See Figure 1 for the PRISMA Flow Diagram of study screening and selection.

### 2.2 The broader molecular PTSD literature umbrella review selection and search strategy

The umbrella review was conducted using the broader molecular PTSD literature, through reviews and meta-analyses on biological markers associated with trauma-exposed individuals who met a clinically relevant threshold for PTSD. For the purpose of this review, the broader molecular PTSD literature included trauma-exposed individuals who did not meet our definition of a first responder. The biomarkers assessed included DNAm, RNA transcription, and biological age as derived from DNAm and RNA transcripts.

The criteria used to select studies from the broader molecular PTSD literature for inclusion in the review were as follows:

- Only secondary research was included
- Only papers published after 2012 were included
- Only human studies were included
- Subjects meeting the definition of first responders were omitted
- The study population must consist of patients who meet a clinically relevant threshold of PTSD, established using standard diagnostic criteria
- The study must measure one or more biomarkers that are included in a model with PTSD
- Cannot incorporate theoretical studies or text and opinion as their primary source of evidence
- Must include methodology for study inclusions
- Must include information on the qualitative or quantitative data found in the original review
- Can include both genetic and epigenetic research, if the data is separated and conclusions are separated
- Both biological marker and PTSD diagnostic criteria must be present for the same individual.

The PubMed, CINAHL, PTSDpubs, and APA PsycInfo literature databases were searched using the key words NOT (“first responders” OR “paramedics” OR “firefighters” OR “police officers” OR “emergency medical technician” OR “nurses” OR “paramedicine” OR “World Trade Center Responders” OR “law enforcement” OR “rescue” OR “nursing” OR “emergency” OR “disaster” OR “search and rescue”) AND (“post-traumatic stress disorder” OR “PTSD” OR “posttraumatic stress disorder” OR “post traumatic stress disorder” OR “post traumatic stress symptoms” OR “PTSS” OR “post-traumatic stress symptoms” OR “acute stress disorder”) AND (“epigenetic*” OR “transcriptom*” OR “mRNA” OR “DNAm” OR “DMAme” OR “transcriptomic clock” OR “EAA” OR “epigenetic age” OR “DNA methylation clock” OR “DNA methylation age” OR “transcriptomic age” OR “DNA methylation” OR “biological embedding” OR “molecular embedding” OR “gene expression” OR “RNA expression” OR “epigenetic clock” OR “GrimAge” OR “PhenoAge” OR “FitAge” OR “Horvath” OR “DunedinPACE” OR “Pace of Aging” OR “biological age”).

**Figure 2:**
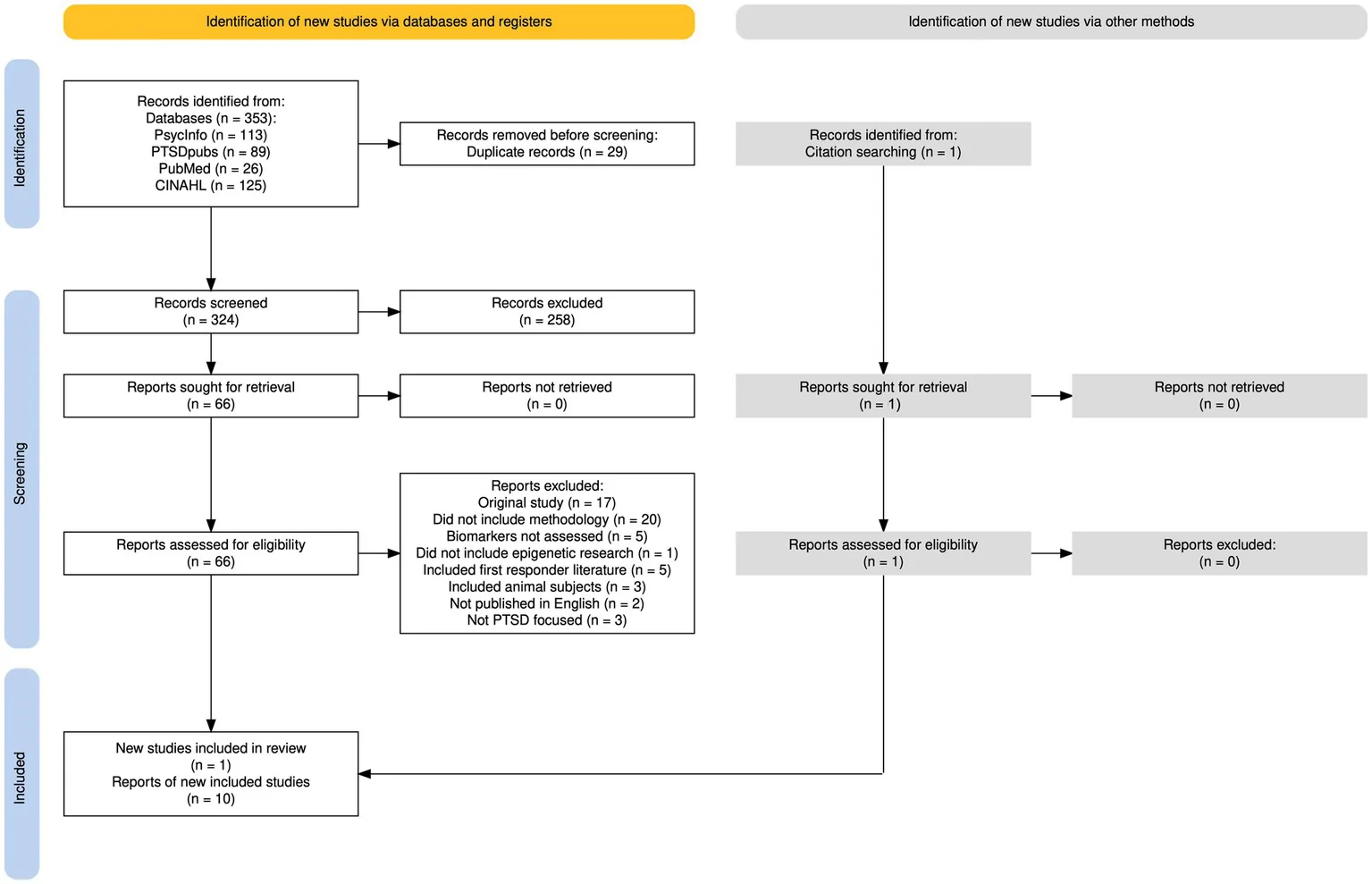
Flow diagram of the selection process of the 11 selected reviews and meta-analyses for the umbrella review on the broader molecular PTSD literature. Each box indicates a step in the flow diagram of the selection process. The right side indicates how many studies were excluded in each step, while the left shows how many papers remained. A total of 353 records were identified, and then 29 duplicates were removed before screening. 258 reports were excluded during the screening process, and then 66 reports were assessed for eligibility. A total of 56 reports were excluded, leading to ten studies being included, with the addition of one record via citation. A total of 11 meta-analyses and reviews were included in the umbrella review for the broader molecular PTSD literature.

See Figure 2 for the PRISMA Flow Diagram of study screening and selection.

### 2.3 Eligibility criteria

Studies published prior to 2012 were excluded from the review due to the wide adoption of the Illumina Infinium HumanMethylation450 BeadChip (450 K array) released mid-2011, which was a significant advancement in epigenetic research in comparison to previously used technologies. Reviews and meta-analyses that included studies prior to 2012 were not omitted, so long as the review itself was published in or after 2012. Literature that compiled results of both first responders and individuals who did not meet our classification of first responders was excluded from the study completely. Only one paper yielded this combined population (Marchese et al., 2022), which included first responders and “nontraditional responders” who do not face true chronic vocational trauma. Studies focused on transgenerational trauma were omitted due to the review focusing solely on individual cell states over time within and across individuals, omitting any form of inheritance besides mitotic.

### 2.4 Study selection and data extraction

Study selections were performed separately, as shown in Figure 1 for first responders and Figure 2 for the broader molecular PTSD literature. The studies were exported to Excel, where they were searched for duplicates. The titles and abstracts were reviewed by CEG and SVM for full-text screening eligibility based on the criteria listed above. Then, the full texts were examined for eligibility and agreed upon by authors CEG and SVM. Data extraction was performed through Excel by two authors, CEG and SVM, and disputes were decided by author SMM.

## 3 Key themes identified

### 3.1 Within the first responder scoping review

The online search yielded a total of 135 results. After removing duplicates and screening abstracts and titles, 103 articles underwent a full reading, and nine were included. The excluded articles were not original studies (*n* = 7), did not focus on first responders (*n* = 84), and did not assess biomarkers (*n* = 3). Of the reviews included, four focused solely on epigenetics, four focused on transcriptomics, and one focused on both epigenetics and transcriptomics. Study characteristics and results are provided in Table 1.

**Table 1:** Included studies on epigenetic and transcriptomic signatures of PTSD in first responders.

Study (year)	Sample size (#)	Sex (% male)	Occupation	Primary exposure	Assessment of PTSD	Tissue sampled	Molecular marker assessed	Example genes annotated	Main findings
Krzyzewska et al. (2018)	73 (PTSD patients: 34; Traumatized Controls: 39)	PTSD patients: 52.9; Traumatized Controls: 51.3	Police Officers	Traumatic Event	DSM-IV criteria for current PTSD, with a CAPS score ≥ 45	Whole Blood	DNA Methylation	*PITX3*; *NEUROD1*; *RET*; *PAX8*	Of the 6 significant enriched pathway-based sets, the lowest *p*-values were involved in the dopaminergic neurogenesis pathway The *PAX8* gene is suggested as a promising candidate for further studies on PTSD
Kuan et al. (2017b)	324 (Current: 81; Past: 42; Never: 201)	100	World Trade Center Responders	World Trade Center Terrorist Attack	SCID; PCL-17	Whole Blood	RNA	*NDUFA1*; *CCDC85B*; *SNORD54*; *FKBP5*; *SNORD46*; *CASP2*; *SOD1*; *BBC3*; C9*orf*84	*FKBP5* was among the topmost overexpressed genes There was a downregulation of *SNORD46*, *NDUFA1*, *CCDC85B*, and *SNORD54* There was an upregulation of *FKBP5* seen in current PTSD relative to never PTSD The polygenic expression model used to compare current, past, and never PTSD groups found the scores were similar in current and past PTSD, with both groups scoring higher than trauma-exposed controls without any history of PTSD PTSD was linked to glucocorticoid receptor signaling, Neural Growth Factor signaling, and several immunity-related pathways to PTSD, but none of the pathways remained significant after FDR correction
Kuan et al. (2021b)	226	100	World Trade Center Responders	World Trade Center Terrorist Attack	PCL-17	Whole Blood	RNA	*IFI17* (interferon-α-inducible protein 27); *AHSP*; *ALAS*; *HMBS*	The top gene associated with total PCL was *IFI17*, a gene involved in innate immunity Avoidance differed the most from other dimensions for DE genes and AS events Findings supported that unique biological pathways may underpin PTSD subtypes DE analyses identified 1, 48, 15, and 49 significant genes that were associated with re-experiencing, avoidance, numbing, and hyperarousal, respectively
Kuan et al. (2021a)	324 (Current PTSD: 81; Past PTSD: 42; Never: 201)	100	World Trade Center Responders	World Trade Center Terrorist Attack	SCID; PCL-17	Whole Blood	DNAm Age (Horvath; Hannum; PhenoAge; GrimAge); Transcriptional Age (RNAAgeCalc)	Not Stated	WTC responders with current PTSD showed accelerated transcriptional aging compared to WTC responders who did not have PTSD WTC responders who had PTSD in the past did not show accelerated aging Transcriptional age acceleration was positively associated with the avoidance dimension of PTSD GrimAge acceleration was significantly associated with PTSD diagnosis PhenoAge, Hannum, and Horvath methylation age acceleration were not reliably related to PTSD GrimAge showed significant age acceleration with PTSD diagnosis, total PCL, avoidance, and numbing dimensions
Toledo et al. (2022)	324 people (Current PTSD: 81; Past PTSD: 42; Never: 201)	100	World Trade Center Responders	World Trade Center Terrorist Attack	PCL-C	Blood	RNA	*SYT1*; *DOCK10*; *SOX5*; C9*orf*84 (*SHOC1*); *NOS1AP*; *TRIM11*; *FKBP5*	Based on differential expression, the past group is more similar to the current group than it is to the never group PTSD appears to be chronic and that psychological interventions are treatments to alleviate the symptoms 12 differentially expressed genes within our data were associated with the neurotrophic signaling pathway A majority of genes in the neurotrophic signaling pathway were underexpressed in PTSD patients, which could contribute to the symptoms experienced by those with PTSD 9 differentially expressed genes involved in the ErbB signaling pathway There was an upregulation of the *SYT1* gene in PTSD cohorts, which interacts with Wnt pathways PTSD shares gene expression patterns with Major Depressive Disorder, Schizophrenia, Parkinson’s Disease, and Alzheimer’s Disease
Kuan et al. (2019)	39 (Case: 20; Control:19)	100	World Trade Center Responders	World Trade Center Terrorist Attack	SCID	Whole Blood	RNA	*FKBP5*; *PI4KAP1*; *PF4*; *REST*; *SEPT4*;	Monocytes showed the largest and most distinct transcriptomic changes in PTSD The upregulation in the candidate gene *PF4* was replicated *FKBP5* and *PI4KAP1* were both upregulated, but the consistent upregulation of *FKBP5* across cell types potentially indicated that the effect of PTSD on *FKBP5* may not be cell-specific It is suggested that gene expression indicative of immune dysregulation is common across several immune cell populations in PTSD
Kuan et al. (2017a)	473 (Current: 171; Past: 100; Never: 202)	100	World Trade Center Responders	World Trade Center Terrorist Attack	SCID	Whole Blood	DNA Methylation	*ZDHHC11*; *CSMD2*	PTSD was associated with pathways underpinned by differentially methylated genes, regulating neuron signaling, inflammation, and multiple aspects of physical health The cGMP-PKG signaling pathway has been identified as the top KEGG pathway for PTSD The second most significant pathway for PTSD was oxytocin signaling Several pathways related to immunity were identified, which supports evidence that PTSD is associated with altered inflammatory processes
Miller et al. (2020)	47	38.3	First-Year Australian Bachelor of Paramedic Science Students	Traumatic Event	PCL-5	Saliva	DNA Methylation	*FKBP5*; *NR3C1*	DNA methylation of different *FKBP5* and *NR3C1* sites was significantly associated with PTSD symptom severity, PTG, and resilience DNA methylation in *FKBP5* site cg07485685 was a predictor of both PTSD symptom severity and resilience in opposite directions One CpG in the *FKBP5* gene, cg07485685, was significantly associated with PTSD symptom severity DNA methylation of promotor-associated region cg03906910 of *NR3C1* was significantly associated with heightened PTSD symptom severity Low PTG after trauma was significantly associated with both *FKBP5* and *NR3C1* DNAm Resilience was significantly associated with DNAm of five sites of *NR3C1* and two sites of *FKBP5*
Mehta et al. (2022)	39	38.5	First-Year Australian Bachelor of Paramedic Science Students	Traumatic Event	PCL-5	Saliva	Epigenetic Age (Horvath Algorithm; GrimAge; SkinBloodAge)	Not Stated	Psychological distress reported pre-fieldwork trauma was significantly associated with epigenetic age acceleration before and after fieldwork trauma Cluster D symptoms were consistently associated with increased epigenetic age acceleration After trauma exposure, burnout and secondary traumatic stress were significantly associated with increased epigenetic aging A stronger sense of organizational membership before trauma exposure was associated with decreased epigenetic aging before and after fieldwork trauma Receiving emotional and instrumental social support after fieldwork trauma was associated with reduced epigenetic aging Epigenetic age acceleration was associated with components of compassion fatigue, such as burnout and secondary traumatic stress

These are the nine studies that were selected for the scoping review for first responders and molecular correlates of PTSD. Data extracted included the sample size, sex, occupation of the participants, primary exposure to trauma, how PTSD was assessed, tissues sampled, the molecular marker assessed, example genes annotated, and the main findings. The full quality assessment is attached as Supplementary material. PTSD, post-traumatic stress disorder; DSM-IV, Diagnostic and Statistical Manual of Mental Disorders 4th Edition; CAPS, Clinician Administered PTSD Scale; SCID, Structured Clinical Interview for DSM-IV; PCL, Posttraumatic Stress Disorder Checklist; DE, differentially expressed; AS, alternative splicing; WTC, World Trade Center; PTG, Post-traumatic growth; DNAm, DNA methylation.

#### 3.1.1 Study characteristics

##### 3.1.1.1 Participants

Of the samples studied, six included only males (Kuan et al., 2017a, 2017b, 2019, 2021a, 2021b; Toledo et al., 2022). Of the studies that reported mean age, the lowest reported mean age was 23.43 years (Miller et al., 2020) and the highest reported mean age was 52.94 years (Kuan et al., 2017b). The countries of origin of the studies were the United States of America (*n* = 6), Australia (*n* = 2), and the Netherlands (*n* = 1). Six studies reported smoking status (Kuan et al., 2017a, 2017b, 2019; Miller et al., 2020; Krzyzewska et al., 2018; Mehta et al., 2022). Two studies focused on first-year bachelor of paramedic science students who were exposed to potentially traumatic events as part of their fieldwork placement (Miller et al., 2020; Mehta et al., 2022). This population met our operational definition of a first responder because the self-reported trauma that was measured, such as exposure to patient deaths, attending suicide scenes, and aggressive patients (Mehta et al., 2022), pertained to their paramedicine field training.

##### 3.1.1.2 Research designs

PTSD ascertainment varied across included studies, and most diagnostic procedures were not equivalent. In one study, PTSD was established using a structured clinician-administered diagnostic interview, the Clinician Administered PTSD Scale (CAPS) according to DSM-IV criteria (Krzyzewska et al., 2018). Three studies trained master’s level psychologists to administer the PTSD module of the Structured Clinical Interview for DSM-IV (SCID) (Kuan et al., 2017a, 2021a, 2017b), and one stated they used the SCID to determine history of DSM-IV World Trade Center PTSD (Kuan et al., 2019). Six studies used versions of the Posttraumatic Stress Disorder Checklist (PCL), a self-reported measure to assess PTSD symptom severity. One study used the PCL-C (Toledo et al., 2022), two studies used the PCL-5 (Miller et al., 2020; Mehta et al., 2022), and three used the PCL-17 to assess PTSD symptom severity of the past month (Kuan et al., 2021a, 2021b), and past week (Kuan et al., 2017b). The timing of PTSD assessment and samples taken were most often concurrent (*n* = 8), but one study did not explicitly state the timing of sample collection (Krzyzewska et al., 2018). The technology used included the Illumina HumanMethylation450 BeadChip (*n* = 2), RNA-Seq (*n* = 5), and Illumina EPIC (*n* = 2). The effect size cutoffs were not explicitly stated in every paper (*n* = 9). The types of studies included were RNA sequencing studies (*n* = 4), candidate gene studies (*n* = 2), clock studies (*n* = 2), and epigenome-wide association studies (EWAS) (*n* = 1).

##### 3.1.1.3 Quality assessment

For the scoping review, the overall quality assessment yielded positive appraisals that led to the inclusion of all intended papers. The two quality thresholds not consistently met included the identification of confounding factors and the presentation of strategies for addressing these confounders. A main quality concern lies with the sample limitations due to the repeated use of the Stony Brook World Trade Center-Health Program. While the Stony Brook World Trade Center-Health Program is a valuable cohort for molecular PTSD research, its repeated use across multiple studies in this review may limit generalizability of the findings due to the non-independence of the included cohorts. The extent of participant overlap is unclear, preventing determination of the total number of unique participants across studies. Full quality assessments for the first responder papers are located in Supplementary Material.

#### 3.1.2 Summary of epigenetic findings

##### 3.1.2.1 The first responder scoping review focused on the impact of PTSD on epigenetic aging and contributing or mitigating factors

Two out of the five studies focusing on epigenetics included epigenetic aging in their studies (Kuan et al., 2021a; Mehta et al., 2022). Kuan et al. (2021a) found that GrimAge acceleration was positively associated with avoidance and numbing PTSD symptoms, while Mehta et al. (2022) supported that increased epigenetic age acceleration was associated with Cluster D PTSD symptoms. Both studies supported the association between increased overall PTSD symptom severity and increased GrimAge acceleration (Kuan et al., 2021a; Mehta et al., 2022). Mehta et al. (2022) revealed that PTSD symptom severity at baseline and follow-up were associated with epigenetic age acceleration. This study took place over a 12-month period, during which the first-year paramedicine students were assessed before their field experience (baseline) and after exposure to a potentially traumatic event as part of their fieldwork placement (post-field experience). In addition, the authors observed that increased epigenetic age acceleration was associated with components of compassion fatigue (secondary traumatization and burnout), which were measured through the Professional Quality of Life Scale: Compassion Satisfaction and Fatigue Version 5 (ProQOL R–V). To combat this, receiving emotional and instrumental social support after fieldwork trauma was associated with decreased epigenetic age acceleration after trauma exposure, while a stronger sense of organizational membership before trauma exposure was associated with decreased epigenetic age acceleration both before and after trauma exposure (Mehta et al., 2022).

##### 3.1.2.2 Limited pathways and markers were identified to be associated with PTSD in the first responder scoping review

Epigenetic and transcriptomic analyses often provide exhaustive lists of individual genes, which can be impractical for interpretation, thus, a standard approach is to summarize the large gene list as a smaller list of more easily interpretable pathways (Reimand et al., 2019). Throughout this manuscript, the included literature often presented pathway-based interpretations, noting replicated genes in these pathways. In the first responder literature, three out of five studies focusing on epigenetics emphasized markers and pathways that were associated with PTSD (Kuan et al., 2017a; Miller et al., 2020; Krzyzewska et al., 2018). Krzyzewska et al. (2018) revealed a possible association between PTSD and the dopaminergic neurogenesis pathway, and the authors stated this was implicated in previous PTSD research due to the pathway’s role in stress reactivity and anhedonia. In addition, this study identified *PAX8* as a candidate for future research and stated the gene was implicated in thyroid and central nervous system (CNS) development and sleep disturbances. Kuan et al. (2017a) associated PTSD with multiple pathways, including several related to immunity, the cGMP-PKG signaling pathway, and the oxytocin signaling pathway. It was also found that DNAm of *FKBP5* and *NR3C1* were significantly associated with low post-traumatic growth, PTSD symptom severity, and resilience (Miller et al., 2020).

##### 3.1.2.3 The first responder scoping review highlighted an inverse relationship between resilience and PTSD symptom severity

One out of five studies focusing on epigenetics studied epigenetic mechanisms underpinning PTSD and resilience (Miller et al., 2020). The authors found that the differential DNAm of *NR3C1* was associated with heightened resilience at two sites and reduced resilience at three sites. The authors claimed that *NR3C1* DNAm being both positively and negatively associated with resilience may reflect how resilient individuals are able to maintain healthy functioning, even when facing continued exposure to distressing events. Additionally, the DNAm of *FKBP5* at site cg07485685 was associated with reduced PTSD symptom severity and heightened resilience, the authors noting that this further supported the HPA-dysregulation hypothesis of PTSD and provided evidence supporting the inverse relationship between PTSD and resilience (Miller et al., 2020).

#### 3.1.3 Summary of transcriptomic findings

##### 3.1.3.1 First responders with current PTSD exhibited increased transcriptional age acceleration

One out of the five studies focusing on transcriptomics included findings on transcriptomic aging in first responders (Kuan et al., 2021a). The authors found that World Trade Center responders with current PTSD showed increased transcriptional age acceleration in comparison to those who never had PTSD. Additionally, transcriptional age acceleration was positively associated with the avoidance symptom dimension of PTSD. It was found that World Trade Center responders who had PTSD in the past, but do not currently, did not show accelerated transcriptional aging. The authors noted that this suggests that biological age may normalize after effective treatment (Kuan et al., 2021a).

##### 3.1.3.2 PTSD was associated with multiple molecular pathways within the first responder scoping review

Three out of five studies focusing on transcriptomics highlighted pathways associated with PTSD (Kuan et al., 2021b, 2017b; Toledo et al., 2022). Toledo et al. (2022) reported that the differential expression of genes involved in the neurotrophic signaling pathway, ErbB signaling pathway, and Wnt pathways could be associated with PTSD symptomology. Although pathways did not remain significant after FDR correction, Kuan et al. (2017b) highlighted that the differentially expressed genes they stated were involved in glucocorticoid receptor signaling and immunity-related pathways may be associated with PTSD. Kuan et al. (2021b) identified the potential of overall unique biological pathways underpinning disorder subtypes, given the patterns of differential expression associated with each of the four dimensions of PTSD.

##### 3.1.3.3 Inflammation and immunity responses were consistently found across the first responder PTSD literature

Four out of the five studies focusing on transcriptomics showed an association between PTSD and inflammation and immune responses (Kuan et al., 2021b, 2017b, 2019; Toledo et al., 2022). Toledo et al. (2022) explored immune pathways that were associated with PTSD as well as other disorders, and confirmed previously identified genes that exhibited direct correlation to PTSD, such as *FKBP5*. Kuan et al. (2017b) identified HPA-axis and immune dysregulation as biological processes underpinning PTSD, highlighting that *FKBP5* was among their topmost overexpressed genes. Another study also extended previous findings on *FKBP5* and found that it was consistently upregulated across all cell types (Kuan et al., 2019). Through analyses of downstream biological pathways enriched by differentially expressed genes, Kuan et al. (2021b) stated their findings were consistent with previous literature, supporting that PTSD is associated with inflammation and the immune response. Additionally, they emphasized that a gene involved in innate immunity, *IFI17*, was their top gene associated with total symptom severity score. Kuan et al. (2019) found that differentially expressed genes in PTSD were enriched in sets related to immune response and inflammation.

##### 3.1.3.4 First responder studies identified a diverse set of biomarkers associated with PTSD

Four out of five studies focusing on transcriptomics identified biomarkers that exhibited strong associations with PTSD (Kuan et al., 2021b, 2017b, 2019; Toledo et al., 2022). Kuan et al. (2019) supported that across multiple cell types, *FKBP5* and *PI4KAP1* remained significant markers that were consistently upregulated, and Kuan et al. (2021b) identified *IFI17* as their top gene associated with PTSD. Additional markers were found to exhibit consistent expression differences, including *FKBP5*, *NDUFA1*, *CCDC85B*, *SNORD54*, and SNORD46 (Kuan et al., 2017b). In assessing biological and pathological pathways and their connection to PTSD, Toledo et al. (2022) identified differentially expressed genes associated with major depressive disorder, such as *DOCK10*, *SOX5*, and *SHOC1*, as well as *NOS1AP*, *TRIM11*, and *NDUFA1*, which are associated with Schizophrenia, Parkinson’s disease, and Alzheimer’s disease, respectively.

##### 3.1.3.5 The first responder scoping review focused on the molecular trajectory of PTSD

Two out of five studies focusing on transcriptomics included findings on the trajectory of PTSD in first responders (Kuan et al., 2017b; Toledo et al., 2022). Toledo et al. (2022) examined three groups: those who currently have PTSD, those who never had PTSD, and those who are not currently experiencing symptoms of PTSD but have had it in the past. In comparing differential gene expression, they found gene expression was more similar between those who do not currently have PTSD but have had it in the past and those who currently have PTSD than those who never had PTSD. In Kuan et al. (2017b), differentially expressed genes were used to create a polygenic expression score, which then showed that polygenic scores were similar in current and past PTSD, with both groups scoring higher than trauma-exposed controls without any history of PTSD.

### 3.2 Within the broader molecular PTSD literature umbrella review

The online search yielded a total of 353 results. After removing duplicates and screening abstracts and titles, 66 articles underwent a full reading, and 10 were included. Through citation searching, a new study was identified, bringing the total number of eligible studies included to 11. In total, 343 articles were excluded. Of the reports that were sought for retrieval, the excluded articles were original studies (*n* = 17), lacked methodology (*n* = 20), did not assess biomarkers (*n* = 5), included first responders literature (*n* = 5), included animal subjects (*n* = 3), were not published in English (*n* = 2), did not include epigenetic research (*n* = 1), and did not focus on PTSD (*n* = 3). Reviews and analyses included systematic reviews (*n* = 5), critical reviews (*n* = 1), and meta-analyses (*n* = 5), published between 2015 and 2025. Collectively, omitting duplicates, they studied a total of 115 studies and 11 cohorts. Of the reviews included, eight focused solely on epigenetics (Pierce and Black, 2023; Watkeys et al., 2018; Wheater et al., 2020; Palma-Gudiel et al., 2015; Snijders et al., 2020; Zhao et al., 2025; Wolf et al., 2018; Katrinli et al., 2022), one focused on transcriptomics (Breen et al., 2018), and two included matched epigenetic and transcriptomic measures (Rajabi et al., 2025; Mehta et al., 2020). Study characteristics and results are provided in Table 2 for reviews and Table 3 for meta-analyses.

**Table 2:** Reviews on epigenetic and transcriptomic signatures of PTSD in other trauma-exposed populations.

Study (year)	Study design	Total number of studies	Number of studies included focusing on PTSD & epigenetics	Population studied	Measures used for PTSD	Tissues sampled
Rajabi et al. (2025)	Systematic review	28	28	Military Personnel; Veterans; Trauma-Exposed Civilians	DSM-5 criteria	Whole Blood; Blood; Saliva; Serum; Post-Mortem Brain
Pierce and Black (2023)	Systematic review	33	33	“Participants were included whose caregivers engaged in substance use while pregnant or survived a traumatic event(s) while pregnant”	PCL-5	Not Stated
Watkeys et al. (2018)	Systematic review	55	6	Adults; Interpersonal Violence Exposed Mothers; Military Personnel; Rwandan Genocide Survivors; Combat Veterans; Children	Semi-Structured Interview; DSM-IV criteria; CAPS; PCL-S; SCL-90; SRIP; PDS; SCID;	Blood; Saliva
Wheater et al. (2020)	Systematic review	60	7	Adults; Interpersonal-Violence Exposed Individuals; Veterans	Not Stated	Blood; Saliva
Mehta et al. (2020)	Systematic review	51	51	General Population; Veterans; Rwandan Genocide Survivors; Holocaust Survivors, Refugees; 9/11 Survivors	Single Scale; CAPS; SCID; PCL; PCL-C; m-PSS; PCL-M; PSS; CPSS; SRIP; CIDI; PPQ; MINI; IES-R; previous diagnosis of PTSD; Medical records/ proxy interviews	Blood; Saliva; Urine; Serum; Monocytes; Post-Mortem Brain Tissue
Palma-Gudiel et al. (2015)	Critical review	23	4	Not Clearly Stated	CTQ; An Index of Traumatic Events; Early Trauma Inventory; Checklist of 36 Traumatic Event Types	Peripheral Blood (leukocytes); Peripheral Blood (T lymphocytes); Peripheral Blood (PBMC); Saliva

These are the six reviews that were selected for the umbrella review for other trauma-exposed populations and molecular correlates of PTSD. The study designs, total number of studies, and the number of studies focusing on PTSD and molecular correlate data were reported. The specific populations studied were listed, as well as the measures used for PTSD and the samples. The quality assessments for each individual review are provided as Supplementary material. PTSD, post-traumatic stress disorder; PTG, post traumatic growth; MRI, Magnetic Resonance Imaging; DSM-5, Diagnostic and Statistical Manual of Mental Disorders-5; CAPS, Clinician-Administered PTSD Scale; PCL, Posttraumatic Stress Disorder Checklist; SCL, Symptom Checklist; SRIP, Self-Rating Inventory for PTSD; PDS, Posttraumatic Diagnostic Scale; SCID, Structured Clinical Interview for DSM-IV; PSS, PTSD Symptom Scale; CPSS, Child PTSD Symptom Scale for DSM-5; CIDI, Composite International Diagnostic Interview; PPQ, Perinatal Post-Traumatic Stress Disorder Questionnaire; MINI, Mini-International Neuropsychiatric Interview; IES-R, Impact of Event Scale-Revised; CTQ, Childhood Trauma Questionnaire Form.

**Table 3:** Meta-analyses on epigenetic and transcriptomic signatures of PTSD in other trauma-exposed populations.

Study (year)	Study design	Total number of cohorts included	Number of cohorts included focusing on PTSD	Names of cohorts included focusing on PTSD	Population studied	Measures used for PTSD	Tissues sampled
Snijders et al. (2020)	Meta-analysis	3	3	MRS; Army STARRS; PRISMO	Military	CAPS; PCL-6; SRIP; PDL-C; DDRI; DEC	Whole Blood; Peripheral Blood
Zhao et al. (2025)	Meta-analysi	7	7	NCPTSD; TRACTS; Army STARRS; MRS; PRISMO; DNHS; AURORA	Veterans; Active Military; Detroit Residents; Trauma Exposed Adults	CAPS; CAPS-5; PCL; CIDI-SC; SRIP; PCL-C; PCL-5	Peripheral Blood
Wolf et al. (2018)	Meta-analysis	9	9	NCPTSD; TRACTS; MRS; Army STARRS; MIRECC-a; MIRECC-b; PRISMO; DNHS; GTP	Veterans; Active Military; Detroit Residents; Trauma Exposed Adults	CAPS-C; CAPS-L, CIDI-C; CIDI-L; SCID-C; SRIP-C; PCL-C; PCL-L;	Peripheral Blood
Katrinli et al. (2022)	Meta-analysis	3	3	MRS; Army STARRS; PRISMO	Military Personnel	Not Stated	Whole Blood
Breen et al. (2018)	Meta-analysis	5	5	MRS; General Medical Community; Veterans Affairs Medical Center PTSD Outpatient Program; Acute Trauma Exposed ER	Emergency Room Trauma; Veterans; Adults with Trauma Exposure; Children	CAPS; DSM-IV Criteria; PSS items;	Isolated leukocytes; whole blood; Isolated CD14 + monocytes; Isolated mononuclear cells; combined

These are the five meta-analyses that were selected for the umbrella review for other trauma-exposed populations and molecular correlates of PTSD. The study designs of each meta-analysis were provided, as well as the total number of cohorts in the meta-analyses and the number of cohorts focusing on PTSD. The names of the individual cohorts studied were listed, as well as the populations that the cohorts studied were included as well. The quality assessments for each individual meta-analysis are provided as Supplementary material. MRS, US Marine Resiliency Study; Army STARRS, US Army Study to Assess Risk and Resilience in Servicemembers; PRISMO, Prospective Research In Stress-related Military Operations; NCPTSD, Longitudinal National Center for PTSD; TRACTS, Translational Research Center for TBI and Stress Disorders; DNHS, Detroit Neighborhood Health Study; AURORA, Advancing Understanding of Recovery after Trauma; MIRECC, Mid-Atlantic Mental Illness Research Education and Clinical Center PTSD Study; GTP, Grady Trauma Project; CAPS, Clinician-Administered PTSD Scale; PCL, Posttraumatic Stress Disorder Checklist; SRIP, Self-Rating Inventory for PTSD; DDRI, Deployment Risk and Resilience Inventory; DEC, deployment experiences checklist; CIDI, Composite International Diagnostic Interview; PSS, PTSD Symptom Scale.

#### 3.2.1 Study characteristics

##### 3.2.1.1 Participants

Of the populations studied, there were 9/11 survivors (*n* = 1), adults (*n* = 3), children (*n* = 2), Detroit residents (*n* = 2), Holocaust survivors (*n* = 1), interpersonal violence exposed mothers (*n* = 1), interpersonal violence exposed individual (*n* = 1), active military personnel (*n* = 6), refugees (*n* = 1), Rwandan genocide survivors (*n* = 2), trauma exposed adults (*n* = 2), trauma exposed civilians (*n* = 1), veterans (*n* = 6), combat veterans (*n* = 1), individuals who presented to the emergency room (*n* = 1), individuals whose caregivers engaged in substance use while pregnant or survived traumatic events while pregnant (*n* = 1), a general population (*n* = 1), and one population that was not clearly stated. Most studies did not clearly state the participants’ ages, one study noted that most individuals were between 20 and 70 years old (Rajabi et al., 2025), and three provided mean ages (Snijders et al., 2020; Wolf et al., 2018; Breen et al., 2018). The number of male and female participants was often not clearly stated. Participants’ countries of origin were only clearly stated in two studies (Snijders et al., 2020; Rajabi et al., 2025), while other studies did not clearly report them or reported ethnicities instead.

##### 3.2.1.2 Research designs

Types of study designs included in the reviews and analyses were longitudinal (*n* = 2), cohort (*n* = 1), case–control (*n* = 1), cross-sectional (*n* = 2), prospective cohort (*n* = 1), and observational (*n* = 1), while others were not reported (Pierce and Black, 2023; Watkeys et al., 2018; Wheater et al., 2020; Palma-Gudiel et al., 2015). Tissue types included whole blood (*n* = 1), blood (*n* = 4), saliva (*n* = 5), serum (*n* = 2), post-mortem brain (*n* = 1), urine (*n* = 1), monocytes (*n* = 1), peripheral blood (leukocytes) (*n* = 1), peripheral blood (T lymphocytes) (*n* = 1), and peripheral blood (PBMC) (*n* = 1).

##### 3.2.1.3 Quality assessment

For the scoping review, the overall quality assessment yielded positive appraisals that led to the inclusion of all intended papers. Four quality thresholds were consistently met. Of the quality thresholds that were not consistently met, the most prominent were the assessment of the likelihood of bias, methods to minimize errors in data extraction, and whether the critical appraisal was conducted by two or more reviewers independently. Full quality assessments for the broader molecular PTSD literature are located in Supplementary Material.

#### 3.2.2 Summary of epigenetic findings

##### 3.2.2.1 Evidence continues to show that trauma exposure impacts epigenetic age acceleration

Two out of ten reviews and meta-analyses focusing on epigenetics drew conclusions from epigenetic clocks, valuable tools used for quantifying discrepancies between biological and chronological aging (Zhao et al., 2025; Wolf et al., 2018). Two studies reported that PTSD symptom severity was associated with increased epigenetic age acceleration, as per Horvath and Hannum metrics (Zhao et al., 2025; Wolf et al., 2018). Zhao et al. (2025) emphasized that although findings of age acceleration were modest and often amount to only fractions of a year, they have the potential to accumulate and have meaningful effects over time. Wolf et al. (2018) also highlighted the need to capture the impact of trauma on contributing to epigenetic age acceleration. It is important to note that the associations between PTSD and epigenetic aging may vary depending on the epigenetic clock used and the populations, tissues, and outcome measures they are trained on (Liu et al., 2020). While epigenetic clocks are not clinical biomarkers, they can be indicators of chronic stress exposure, such as life trauma and post-traumatic stress (Lim et al., 2022), and are increasingly used to potentially capture the biological embedding of adversity. The first generation of clocks, such as the Horvath (2013) and Hannum et al. (2013) were trained to predict age, providing an epigenetic age estimate that could then be compared to their chronological age, while the second generation, such as DNAm PhenoAge (Levine et al., 2018) and DNAm GrimAge (Lu et al., 2019), were trained on mortality and health indicators to predict length of life and health rather than age (Crimmins et al., 2024).

##### 3.2.2.2 Inflammatory pathways and immune system dysregulation exhibit a consistent molecular association with PTSD

Over half of the included reviews and meta-analyses focusing on epigenetics conceptualized PTSD to involve immune dysregulation (Palma-Gudiel et al., 2015; Snijders et al., 2020; Wolf et al., 2018; Katrinli et al., 2022; Rajabi et al., 2025; Mehta et al., 2020). In their first meta-analysis, Katrinli et al. (2022) found that higher post-traumatic stress symptoms (PTSS) were associated with lower DNAm levels in *F2R*, *CNPY2*, *BAIAP2L1*, and *TBXAS1*. The authors noted that these genes are supported by previous research to be involved in inflammation, immune response, and oxidative stress pathways. Another study highlighted evidence supporting that susceptibility to psychological stress and possibly an overall increased risk of PTSD may be influenced by alterations in the DNAm of genes potentially involved in immune responses and inflammatory processes, such as *CD55, DAZAP2, AHRR, CDC42BPB, DOCK2, EP300*, and *P2RX6* (Rajabi et al., 2025). The association between PTSD and the immune system was further supported by the differential DNAm of CpGs in *AHRR*, which was mentioned to potentially cause excessive immune response and cytokine release, contributing to the development and progression of PTSD symptoms (Rajabi et al., 2025). Mehta et al. (2020) also showed that the DNAm of key inflammatory genes, such as *IL2RA* and *IL18*, was significantly associated with PTSD symptoms. The immune system was shown to not only impact PTSD symptomology and susceptibility but also noted as possibly playing a role in the loss of synchronicity between one’s chronological and epigenetic age (Wolf et al., 2018). Additionally, inflammatory responses were shown to be associated with stress response genes such as *FKBP5* and *NR3C1*, especially at exon 1F, which are commonly privileged in EWAS analyses and found consistently across the literature (Palma-Gudiel et al., 2015; Mehta et al., 2020).

##### 3.2.2.3 Post-mortem brain samples are needed to validate epigenetic findings on PTSD

Three out of ten reviews and meta-analyses focusing on epigenetics linked epigenetic changes in PTSD with alterations associated with brain structure and functioning; however, none utilized direct tissue-based molecular evidence, such as post-mortem brain samples (Wheater et al., 2020; Snijders et al., 2020; Katrinli et al., 2022). Instead, they used indirect methods to draw correlations, such as brain imaging through MRIs (Wheater et al., 2020), or external databases, such as the Blood Brain DNA Methylation Comparison Tool (Snijders et al., 2020) or the IMAGE-CpG database (Katrinli et al., 2022). The analysis of biological tissue, such as post-mortem brain samples, allows for the direct identification of underlying epigenetic and transcriptomic mechanisms in the primary tissue of interest (Jaffee, 2016), which otherwise can only be inferred indirectly when using imaging measures or database approaches. In attempting to observe if the DNAm changes in blood reflect changes in the brain, Snijders et al. (2020), using a publicly available database, found a correlation between DNAm levels in three PTSD associated DMPs and the DNAm levels in the prefrontal cortex, entorhinal cortex, superior temporal gyrus, and cerebellum. It should be noted that the authors emphasized the need to use post-mortem brain samples to confirm these findings (Snijders et al., 2020).

##### 3.2.2.4 Altered DNA methylation was observed in distinct epigenetic markers in relation to resilience and PTSD trajectories

Three out of ten reviews and meta-analyses focusing on epigenetics reported findings on resilience and protective factors in relation to PTSD (Pierce and Black, 2023; Rajabi et al., 2025; Mehta et al., 2020). Rajabi et al. (2025) highlighted one study that linked PTSD to altered DNAm in linoleic acid metabolism genes such as *PLA2G4A*, *PLA2G4B*, *PLA2G1B*, and *ALOX15*, which were also noted to impact stress resilience, memory, and immune regulation. Mehta et al. (2020) stated that in studies that classified resilience as individuals without PTSD, differential DNAm patterns were identified in stress genes like *FKBP5*, immune genes like *MHC* class III, and DNAm maintenance genes like *DNMT3A* and *DNMT3B* (Mehta et al., 2020). Katrinli et al. (2022) found that the CpGs in *F2R*, *BAIAP2L1,* and *TBXAS1* showed only a small change in pre-deployment to post-deployment DNAm. Although the exact time elapsed between pre-deployment and post-deployment assessment was not clearly stated in this paper, the authors noted these findings support their potential as relatively stable biological markers. Pierce and Black (2023), identified protective factors that may alter the expression of these epigenetic markers and mitigate the impacts of traumatic stress, such as secure attachment relationships, engaging in physical exercise, maintaining a healthier diet, and reconnecting with friends and loved ones.

##### 3.2.2.5 Epigenetic research showed limited consideration of sex differences

Nine out of ten reviews and meta-analyses focusing on epigenetics did not acknowledge how sex can shape epigenetic responses to stress. Wolf et al. (2018) observed that, on average, women showed decreased epigenetic age acceleration relative to men; however, they cautioned that it is not known what underlying sex difference this pattern could reflect.

##### 3.2.2.6 *NR3C1* and *FKBP5* were consistently highlighted throughout the broader molecular PTSD literature and discussed as potential biomarkers of treatment response

Four out of ten reviews and meta-analyses focusing on epigenetics addressed therapy and treatment response in relation to PTSD (Watkeys et al., 2018; Wheater et al., 2020; Rajabi et al., 2025; Mehta et al., 2020). Watkeys et al. (2018) highlighted that one study included in their review found that individuals who did not respond to therapy exhibited reduced DNAm of exon-1F of *NR3C1* in comparison to individuals who did respond. *FKBP5* and *NR3C1* were two of the five candidate genes studied in Wheater et al. (2020), and the authors noted that these genes were selected because they are key components of cortisol signaling, which is a pathway of relevance to PTSD. Mehta et al. (2020) and Rajabi et al. (2025) highlighted *FKBP5* as a potential biomarker for treatment or monitoring treatment response, given that the research supports that it contributes to PTSD pathophysiology. Rajabi et al. (2025) supported that some miRNAs, another biomarker, represent potential diagnostic or therapeutic biomarkers for veterans with PTSD; however, implementing these diagnostic and therapeutic methods presents certain challenges, such as heterogeneity in molecular pathways and immune responses, which limit generalizability. They further emphasized the need to use more effective treatments, such as personalized therapies tailored to an individual’s molecular profile. The potential of non-invasive treatments, such as exercise and lifestyle interventions, was discussed but not tested. They also supported that methylation patterns in *NR3C1* may enable early identification of PTSD risk or onset, particularly in trauma-exposed populations (Rajabi et al., 2025).

#### 3.2.3 Summary of transcriptomic findings

##### 3.2.3.1 Inflammation and immunity remain prominent transcriptomic signatures

All three reviews that examined transcriptomic data consistently found altered expression in genes related to immune dysregulation to be the most reproducible transcriptomic signature associated with PTSD (Breen et al., 2018; Rajabi et al., 2025; Mehta et al., 2020). Breen et al. (2018) supported previous findings of immune dysregulation in PTSD, stating that across sex and trauma types, peripheral blood gene expression provided evidence for shared inflammatory profiles and potentially, an overall broader stress response system. Rajabi et al. (2025) reinforced that the upregulation of genes that play a crucial role in immune modulation, such as *IL-10RB, IL-16, and IL-4R,* was shown to lead to inflammatory disinhibition and immune dysregulation in combat-induced PTSD. Two studies further emphasized the consistent differential expression of pro-inflammatory genes such as *CCL4, NF-κB, TNF-α,* and *IL-6* and highlighted the role of systemic inflammation in PTSD (Breen et al., 2018; Rajabi et al., 2025). Mehta et al. (2020) highlighted the importance of using longitudinal studies, expressing that the reliance on cross-sectional studies in available literature makes it unclear whether an elevated immune response precedes or is affected by PTSD.

##### 3.2.3.2 Trauma-specific and sex-specific profiles remain important aspects of PTSD

One out of the three transcriptomic studies explicitly assessed whether sex and trauma type influence molecular pathways in PTSD and found meaningful subgroup variation in sex-specific and trauma-specific profiles (Breen et al., 2018). Three distinct gene expression differences were highlighted, including the downregulation of a wound-healing module in males with a history of combat trauma, upregulation of two modules implicated in lipid processes and MAPK activity in women exposed to interpersonal violence, and one IL-12-mediated signaling module upregulated in men exposed to interpersonal-related trauma. Although subgroups had unique patterns of expression, the authors noted that there still remained considerable overlap in biological pathways impacted, such as the cytokine, innate immune, and type I interferon pathways (Breen et al., 2018).

##### 3.2.3.3 PTSD was associated with stress-regulation pathways

Across reviews, authors highlighted the stress response pathway, providing evidence that transcripts of genes such as *NR3C1* and *FKBP5* frequently appeared as transcriptomic signatures associated with PTSD (Rajabi et al., 2025; Mehta et al., 2020). Mehta et al. (2020) emphasized that differential gene expression in glucocorticoid and immune-regulating genes in military personnel prior to deployment was a predictor of PTSD. Rajabi et al. (2025) highlighted the importance of the *NR3C1* gene in the stress pathway. Authors noted that if the gene’s role in the feedback system is compromised through decreased expression or altered methylation, this could potentially lead to persistent hyperactivation or hypoactivation of the HPA axis, both of which are linked to PTSD pathophysiology.

##### 3.2.3.4 BDNF was highlighted as a prominent transcriptomic marker in relation to resilience

Two studies privileged *BDNF* and discussed their findings on resilience (Rajabi et al., 2025; Mehta et al., 2020). It was reported that high resilience was associated with the downregulation of *CTRA* gene expression, while reduced stress resilience was associated with the overexpression of dopamine-related genes and *BDNF* (Mehta et al., 2020). Rajabi et al. (2025) identified that the downregulation of pathways that are activated by bdnf, such as the phospholipase C-gamma, PI3K, and MAPK/ERK pathways, was associated with reduced resilience and impaired recovery in PTSD.

##### 3.2.3.5 The current state of transcriptomics as a biomarker of PTSD shows promise, but further research is needed

Two studies investigated the current state of diagnostic tools at the time of their publication (Breen et al., 2018; Mehta et al., 2020). Breen et al. (2018) assessed the utility of blood-based diagnostic classifiers through machine learning and found moderate-to-low classification accuracies on independently withheld test data, which reached only 60–65%. Mehta et al. (2020), published two years after, found that patterns of transcriptomic signatures are sufficiently distinct in PTSD to allow machine learning classifiers to predict future PTSD. One of the studies included in their review observed that classifiers accurately predicted PTSD status within their select population 80–84% of the time, and another study reported their classifiers predicted PTSD status four months post-trauma with 88% accuracy within their specific study population.

## 4 Discussion

The primary aim of this review was to contextualize the first responder findings within the broader molecular PTSD literature. The first responder findings highlighted important characteristics of PTSD, such as a broader systemic vulnerability that can lead to comorbid diagnoses, as well as social factors that could positively impact resilience or serve as protective factors against PTSD. These results also revealed key methodological differences across the two bodies of literature that limit the ability to draw definitive epigenetic and transcriptomic distinctions between the populations. This section explores the findings of the first responder scoping review, contextualizing it within the broader molecular PTSD literature umbrella review while highlighting the methodological constraints that impacted cross-study comparisons.

### 4.1 Findings of the first responder scoping review

#### 4.1.1 PTSD in first responders is commonly accompanied by co-occurring psychological and behavioral conditions through shared biological pathways

PTSD rarely occurs in isolation, and individuals often face comorbid diagnoses of anxiety, depression, substance use disorders, or adjustment disorder, as well as nondiagnosable conditions including problematic anger, sleep problems, and hazardous alcohol use (Arjmand et al., 2024). Although both bodies of work acknowledged that PTSD frequently co-occurs with other conditions, the broader molecular PTSD literature often focused on the disorder in isolation, while the first responder literature consistently examined comorbidities and identified shared epigenetic and transcriptomic patterns. The findings from the first responder population suggested overlapping molecular pathways across psychological and neurodegenerative disorders, as well as metabolic conditions. Although comorbidities were not a main focus of the reviewed broader molecular PTSD literature, findings across the two reviews converged in suggesting that PTSD may involve overlapping molecular mechanisms that potentially contribute to vulnerabilities across multiple conditions.

#### 4.1.2 Persistent but modifiable molecular effects of PTSD in first responders can extend beyond symptom remission

Throughout the first responder literature, many studies attempted to understand the longevity of biological effects over time. Toledo et al. (2022) supported the idea that biological embedding could persist beyond symptomatic recovery, demonstrating that biomarkers could potentially be independent of an individual’s presenting symptoms. The findings of Kuan et al. (2017b) supported a future emphasis on biological embedding to help researchers better understand the risk first responders face when returning to work, including the potential reemergence of PTSD symptoms despite the previous absence of outward clinical presentation. These findings suggest that molecular profiles associated with prior PTSD may persist beyond symptomatic remission, potentially serving as biomarkers of vulnerability to symptom recurrence among first responders who remain in occupations characterized by continued trauma exposure. Although the included literature aimed to emphasize the potential lasting impacts of biological embedding, it is important to note that these modifications are neither deterministic nor permanent. Epigenetic and transcriptomic processes are dynamic and responsive to environmental, psychosocial, and therapeutic influences.

#### 4.1.3 The first responder scoping review examined occupational influences on the association between trauma and biological aging

Although both populations demonstrated associations between PTSD and biological age acceleration, the first responder literature framed their findings in a more occupational context in comparison to the reviewed broader molecular PTSD literature. In focusing on protective factors in their occupation, research on first responders found that social support, reduced burnout, and a strong sense of organizational belonging were linked to decreased epigenetic age acceleration. In the reviewed broader molecular PTSD literature, Zhao et al. (2025) suggested that increased epigenetic age acceleration may be especially impactful for younger veterans; however, this also inversely implies an extended window for biological recovery. Younger veterans, and young first responders, may have more time to mitigate or reverse the changes, showing that biological age acceleration should be understood as a modifiable process rather than a fixed outcome. Biological clocks will remain useful in future research when assessing the trajectory of biological age acceleration, especially in first responders who potentially face traumatic experiences for extended periods of time.

### 4.2 Critical gaps impacted the ability to distinguish the first responder scoping review from the broader molecular PTSD literature umbrella review

Although the first responder scoping review provided valuable insight, it also revealed critical gaps that impacted the ability to distinguish the two populations. Three of the most prominent limitations were the inadequate assessment of chronicity, the limited inclusion of diverse samples and female participants, and the overreliance on narrowly defined trauma cohorts. These limitations restricted the field’s ability to fully capture the cumulative nature of trauma exposure in first responders and impacted the generalizability of conclusions.

#### 4.2.1 The first responder scoping review did not assess chronicity at a molecular level, a defining aspect of PTSD within the population

All individuals who experience trauma have the potential to be repeatedly exposed and it is expected that first responders are likely to face an accumulation of multiple traumas over time while in the line of duty. Although chronic occupational trauma is a defining feature of PTSD for many first responders, much of the reviewed literature failed to examine longitudinal exposure and did not explore the impact of compounding trauma as a result of their occupation. Many studies did not model cumulative exposure and rather focused on single high-impact events, such as the 9/11 terrorist attacks. The omission of chronicity impacted researchers’ ability to truly capture first responders’ experiences with PTSD, as supported by the allostatic load theory, which highlights the importance of taking into account the cumulative burden of chronic stress and life events (Guidi et al., 2021). In addition to cumulative trauma, first responder literature also did not address the multifactorial exposure they face in their occupation. Studies often did not survey the compounding impacts of harmful environmental agents uniquely faced by first responders, such as exposure to airborne pollutants due to vehicular traffic, riot control agents, self-defense spray, and lead at firing ranges (Magrini et al., 2014), which may impact epigenetic and transcriptional data. The cumulative and multifactorial nature of occupational exposures among first responders potentially contributes to their unique variations in PTSD presentation and its molecular correlates, which is why future studies in this field must account for chronicity in this population.

#### 4.2.2 Underrepresentation of female subjects impacted sex-specific molecular conclusions

In both bodies of literature, sex differences remained not only underexplored but ignored due to the omission of female participants. This was most commonly seen in the first responder literature, in which only three studies included females in their sample (Miller et al., 2020; Krzyzewska et al., 2018; Mehta et al., 2022). Collectively, all studies focusing on the 9/11 terrorist attacks utilized the Stony Brook World Trade Center-Health Program, and within this program, less than 10% of responders were female. Although female participant data was collected and available, the authors decided to omit this data, stating this exclusion is due to females showing notably different gene expression patterns from males. This drastically impacted the sex specific conclusions in the first responder literature and diminished the representation of females in the first responder population. While the reviewed broader molecular PTSD literature better represented female subjects, samples often included mixed but inconsistently reported sex distributions. While this allowed the individual studies to produce sex specific associations, many were unable to interpret their findings. One study observed sex-specific differences in epigenetic age acceleration, suggesting that, on average, women had decreased epigenetic age acceleration in comparison to men in both Hannum and Horvath indices. The authors noted that the differences could show underlying different rates of aging, differential susceptibility to epigenetic age acceleration, or potential biases in the accuracy of the age algorithms, in which they are more accurate in one sex (Wolf et al., 2018). In addition, a similar theme of underreporting was seen with ethnicities in which there was often a lack of diversity in studies, or ethnic backgrounds were simply not reported. This not only limits generalizability but also impacts the studies because they fail to consider an important factor in epigenetic and transcriptomic research.

#### 4.2.3 The first responder scoping review focused heavily on the molecular impact of the 9/11 terrorist attacks, limiting generalizability to other exposure types

With a substantial portion of the first responder literature focusing on the Stony Brook World Trade Center-Health Program, the strength and generalizability of findings were limited, as the studies were essentially examining variations within a single cohort rather than independent samples. The overlap among the first responder cohorts highlighted how these papers are not independent bodies of evidence, and thus, there are implications for replication and generalizability. Additionally, the focus on the 9/11 terrorist attacks classifies the aims of those studies as defining the impacts of the 9/11 terrorist attacks, rather than the impacts of the overall first responder occupation. This disproportionate focus on one single amassed exposure restricts the diversity of experiences in this population and reduces the validity of findings for broader occupational contexts. In contrast, the reviewed broader molecular PTSD literature was far more diverse, incorporating multiple populations and trauma types, which then could account for greater variability in epigenetic and transcriptomic findings. Additionally, first responder studies often did not survey the type of trauma exposure, providing only limited descriptions of occupational trauma without detailed characteristics. Collectively, these highlight the need to collect new samples of first responders to capture a broader range of trauma exposure and to strengthen external validity.

### 4.3 Consistent findings across trauma-exposed populations

Despite differences in methodology and population characteristics, the literature consistently converged on shared biological mechanisms underlying PTSD and shared a focus on protective factors impacting resilience.

#### 4.3.1 Most studies emphasized commonly identified pathways in molecular literature, while emerging biomarkers were not replicated

Across both reviews, immune dysregulation and stress response pathways were consistently identified as central biological mechanisms underlying PTSD. Although this reinforced the potential involvement of inflammation and altered HPA-axis regulation in the development and progression of PTSD, specific biomarkers within these pathways varied substantially. Often, individual studies identified unique DNA methylation sites and transcripts that did not replicate across the other available literature. Of the biomarkers that were replicated, many were privileged in prior PTSD research or identified through candidate gene approaches. Most often, studies focused on the differential DNAm and expression of *FKBP5* and *NR3C1,* associating these biomarkers with PTSD severity, resilience, and post-traumatic growth trajectories. This highlights the need to focus on replicating new biomarkers and shifting the focus away from those that are consistently privileged to expand the recognized epigenetic and transcriptomic alterations associated with PTSD.

#### 4.3.2 Both reviews prioritized protective factors but differed on their definition of molecular resilience

Across both bodies of literature, there is a shared emphasis on utilizing epigenetic and transcriptomic markers as biologically informed approaches to identify risk and to guide targeted prevention strategies for PTSD. Individual studies in both the scoping and umbrella review highlighted important factors that can help potentially buffer the impact of occupational or general trauma, such as physical activity, social support, and workplace culture. However, key differences between the two literatures arise in how resilience and protection are framed and prioritized. The first responder literature primarily centered current and cumulative occupational trauma, while the reviewed broader molecular PTSD literature incorporated more heterogeneous influences, including Adverse Childhood Experiences (ACEs) (Felitti et al., 1998) and variations in trauma type, resulting in a more diverse biological imprint. Additionally, there are distinctions in the conceptualization of resilience in which first responder literature views resilience as an active process that influences PTSD trajectories, while the reviewed broader molecular PTSD literature more commonly defines resilience as the absence of the disorder. These findings demonstrate that while both literatures prioritize resilience and protective factors, they diverge in their conceptual focuses and framing, reflecting the distinct contexts in which trauma is experienced.

### 4.4 Methodological limitations impacting cross-study comparisons

The methodological inconsistencies across both bodies of literature affected the ability to compare the two populations and raised concerns. The inadequate control of major confounders and the variability in tissue sources limited biologically meaningful conclusions and call for a deeper understanding of factors that can impact epigenetic and transcriptomic data.

#### 4.4.1 Most studies did not address smoking as a major confounder while highlighting canonical smoking genes

A notable methodological concern was the insufficient consideration of smoking, given its well-established epigenome-wide impact on DNAm. The prevalence of smoking among those with PTSD highlights the importance of addressing its impact, with one systematic review indicating that those with PTSD are twice as likely to smoke (Kearns et al., 2018). Canonical smoking genes such as *AHRR* and *DOCK2* were replicated in many studies, yet they were not used to explore the relationship between PTSD and the prevalence of smoking. Smoking is one of the most well validated effects outside of disease diagnosis (Joehanes et al., 2016; Zeilinger et al., 2013), yet many studies did not investigate this association and only occasionally reported smoking status. Additionally, molecular studies on PTSD are subjected to other confounding influences, including sleep disturbances, comorbid medical and psychiatric disorders, medication, illicit drug use, and differences in the timing of biological sample collection (Lind et al., 2019; Schmidt et al., 2013; Sharp et al., 2026). An additional confounder is medication-associated weight gain, which may affect the direct relationship between PTSD and obesity (Masodkar et al., 2016). The call for a focus on addressing smoking within PTSD molecular literature can be complicated by collider-confounder bias due to the potential bidirectional relationship between smoking, PTSD, and molecular measures (Cole et al., 2010). Future studies should note the potential collider or confounder association among PTSD, smoking, and molecular measures when attempting to disentangle the examination of the disorder in this population.

#### 4.4.2 Cross-study comparisons were greatly impacted by tissue-specificity

Across the field, there is a strong consensus that epigenetic and transcriptomic findings are highly tissue-specific (Roadmap Epigenomics Consortium, 2015; Yanai et al., 2005), and thus, cross-tissue comparisons must be interpreted with caution. Given that tissue types varied widely across studies and within reviews and analyses, cross-study comparisons between first responders and the broader molecular PTSD literature became complicated. Cross-tissue comparisons were seen in the reviews and analyses of the broader molecular PTSD literature, where many studies ignored the importance of tissue-specific findings. A highlighted issue was that numerous studies drew conclusions on brain functioning and neural plasticity based on DNAm or gene expression data derived from blood or saliva without incorporating brain tissue samples. Of the papers that did use this method, they concluded that studies utilizing post-mortem brain samples must be used to confirm these findings, agreeing on the importance of tissue-specificity.

### 4.5 Suggestions for future research on first responders and trauma-exposed individuals

#### 4.5.1 Trauma literature should prioritize longitudinal designs to capture chronicity and reoccurrence in molecular correlates of PTSD

Based on the reviewed literature, several key recommendations emerged that could potentially enhance research of the first responder and the overall broader molecular PTSD literature. A primary area of improvement is to better address the chronicity and recurrence of trauma exposure seen in the first responder population. In understanding an individual’s experience with PTSD, such as a first responder, individual who experienced interpersonal violence, or military service member, it is important to determine if they underwent a singular traumatic event or have faced recurring traumatic events. Future studies on first responders should prioritize longitudinal designs to examine the cumulative burden of repeated trauma over one’s career, allowing for more robust epigenetic and transcriptomic analyses. A similar suggestion can be made for the broader molecular PTSD literature, in which a major shift towards longitudinal study designs can examine the impact of trauma exposure and its progression over time. Examining biological embedding as a longitudinal process would benefit overall trauma literature and improve our understanding of the trajectory, risk factors, changes in symptomology, and treatment responses for individuals with PTSD.

#### 4.5.2 Further research is needed on how DNAm profiles may predispose individuals to PTSD reemergence after symptomatic recovery

The repeated trauma exposure faced by first responders in the line of duty also calls for a greater focus on biological embedding and its course over time. Specifically, an emphasis should be put on determining if this molecular profile could potentially prime individuals for latent trauma responses after appearing to no longer exhibit measurable symptoms of PTSD. This can allow researchers to better understand the longitudinal impact of trauma, whether from occupational experiences in first responders or early life adversities faced by trauma-exposed populations. Examining the prevalence of symptom reemergence and the continuing epigenetic and transcriptomic markers even after recovering from PTSD could potentially provide valuable insight into the potential long-term vulnerability of the disorder.

#### 4.5.3 Future research should address epigenetic and transcriptomic modifications in response to treatment

The literature reviewed did not include studies that administered treatment or evaluated the biological effects of non-invasive interventions on PTSD. Developing an understanding of how epigenetic and transcriptomic mechanisms respond to treatment could possibly offer an objective measure of therapeutic effectiveness. This is particularly relevant for the first responder population, who have been shown to underreport mental health symptoms (Marshall et al., 2021), potentially affecting the accuracy of self-reported data. Future research should focus on examining the epigenetic and transcriptomic effectiveness of treatments and utilize these biological tools as objective measures of responsiveness to treatment.

#### 4.5.4 Epigenetics and transcriptomics may be useful to quantify the impacts of psychological trauma in first responders and other trauma-exposed individuals

Epigenetic and transcriptomic biomarkers hold the potential to inform precision medicine, but while current research supports their utilization in experimental settings, further development is needed before being introduced as reliable clinical classifiers. Overall, the application of existing PTSD classifiers to first responder populations remains unclear, as they have been developed in cohorts exposed to a wide variety of trauma exposures rather than the characteristic chronic and repeated occupational trauma experienced by first responders. The use of biomarkers within the first responder community may serve as a valuable method for quantifying the biological impact of trauma. Given the persistent stigma surrounding mental health within this community, the framing of information in terms of measurable biological changes rather than solely psychological effects may support more effective communication between clinicians and patients. Finally, future research should address whether populations of interest are receptive to biological testing. Some individuals may be reluctant to provide biological samples or prefer not to know their potential biological risk for developing mental health conditions. Although epigenetic and transcriptomic data remain valuable to the research community, it’s essential to evaluate their application to the general population and whether they perceive it as beneficial or burdensome.

## 5 Limitations

The current review was subject to several key limitations that should be considered when interpreting findings. Firstly, the available literature on first responders showed limited sample diversity with respect to sex, type of trauma exposure, and the populations and cohorts studied. Notably, a substantial portion of first responder studies focused on a single cohort examining the 9/11 terrorist attack, which does not represent the broader first responder population and the range of occupational trauma exposures they encounter within the field. Additionally, cross-population comparisons were constrained by the significant heterogeneity in tissue samples and the lack of clinical PTSD diagnosis within the first responder literature. No randomized control trials were identified, and a majority of studies were quasi-experimental. Additionally, the lack of longitudinal study designs restricted the ability to examine the impact of trauma on biomarkers over time. All of the trauma-exposed literature combined the populations together in their analysis, limiting our ability to draw conclusions on specific populations, such as veterans and active military personnel. Finally, the existing literature relied on candidate gene approaches and often focused on previously identified targets, limiting the discovery of potentially relevant biomarkers underlying PTSD.

## 6 Conclusion

In conclusion, the current state of available epigenetic and transcriptomic research inadequately characterizes the molecular underpinnings of PTSD in both the first responder population and the broader molecular PTSD literature. Due to this, it is difficult to discern if there is a true lack of convergence among trauma-exposed populations, complicating the ability to distinguish one population from a generalizable signal. The absence of clear molecular differences may reflect contextual factors, individual or population variability, statistical power, or methodological and technological constraints, rather than a true lack of biological distinction between these two populations. Future research should prioritize longitudinal designs that capture the chronic occupational trauma in first responders, incorporate more diverse samples, and utilize standardized methods to better replication and comparisons across studies. There is a need to move beyond candidate gene approaches and consistently used cohorts to better capture the complexity of our biological systems and allow for novel discoveries that could advance our understanding of the long-term molecular and health consequences of trauma in the first responder population. With the appropriate methodology and improvements to study design and representation, we can better our understanding of which aspects of PTSD become biologically embedded over time. This may provide insight into how trauma related biological impacts can influence symptom progression, vulnerability, and treatment response for all those who have experienced trauma.

## Data Availability

The original contributions presented in the study are included in the article/Supplementary material, further inquiries can be directed to the corresponding author.
