# Supplementary material for: Comparing the molecular correlates of PTSD in first responders and the broader molecular PTSD literature: a hybrid review
Source: Front Psychol. 2026 Aug 20;17:1875411. doi: 10.3389/fpsyg.2026.1875411 (PMC13543159; doi:10.3389/fpsyg.2026.1875411)
Supplement: Supplementary file 1 [file Table_1.pdf]

Study (year)	Sample Size (#)	Mean Age (years)	Sex (% male)	Race/ Ethnicity (%)	Occupation (#)	Country	Primary Exposure	Assessment of PTSD	Smoking Status (%)	Tissue Sampled	Molecular Marker Assessed	Timing (assessment of PTSD and sample taken)	Technology Used	Effect Size Cutoff	Threshold of Significance	Type of Study	Differential Molecular Markers	Example Genes Annotated	Main Findings	Quality Assessment
Krzyzewska et al. [39]	73 (PTSD patients: 34; Traumatized Controls: 39)	PTSD patients: 41.45; Traumatized Controls: 40.15	PTSD patients: 52.9; Traumatized Controls: 51.3	Dutch (100)	Police Officers (73)	Netherlands	Traumatic Event	DSM-IV criteria for current PTSD, with a CAPS score ≥ 45	Current Smoker (PTSD patients: 21.9; Traumatized Controls: 20.5)	Whole Blood	DNA Methylation	Not Explicitly Stated	Illumina HumanMethylation450 BeadChip	Not Stated	Genome-wide significance by FDR q < 0.05 for DMPs, and FWER-adjusted P < 0.05 for DMRs	Candidate Gene Study	6 significant enriched pathway-based sets; two significant enriched GO terms; 1 observed consistent hyper methylation	*PITX3; NEUROD1; RET; PAX8*	•Of the 6 significant enriched pathway-based sets, the lowest p-values were involved in the dopaminergic neurogenesis pathway<br>•The *PAX8* gene is suggested as a promising candidate for further studies on PTSD	Include
Kuan et al. [35]	324 (Current: 81; Past: 42; Never: 201)	Current: 52.94; Past: 51.57; Never: 51.36	100	Caucasian (Current: 85.2; Past: 78.6; Never: 90.0); Other (Current: 14.8; Past: 21.4; Never: 10.0)	World Trade Center Responders (324)	United States of America	World Trade Center Terrorist Attack	SCID; PCL-17	Non-Smoker (100)	Whole Blood	RNA	Concurrently	RNA-Seq	Not Explicitly Stated	(FDR) < 0.05 in the discovery cohort and nominal p-value < 0.05 in the replication cohort	RNA Sequencing Study	99 significant genes were differentially expressed	*NDUFA1; CCDC85B; SNORD54; FKBP5; SNORD46; CASP2; SOD1; BBC3; C9orf84*	•*FKBP5* was among the topmost overexpressed genes<br>•There was a downregulation of *SNORD46, NDUFA1, CCDC85B* , and *SNORD54*<br>•There was an upregulation of *FKBP5* seen in current PTSD relative to never PTSD regardless of the genotypes<br>•The polygenic expression model used to compare current, past, and never PTSD groups found the scores were similar in current and past PTSD, with both groups scoring higher than trauma-exposed controls without any history of PTSD<br>•PTSD was linked to glucocorticoid receptor signaling, Neural Growth Factor signaling, and several immunity-related pathways to PTSD, but none of the pathways remained significant after FDR correction	Include
Kuan et al. [34]	226	52.67	100	Caucasian (84.5)	World Trade Center Responders (226)	United States of America	World Trade Center Terrorist Attack	PCL-17	Not Stated	Whole Blood	RNA	Can Be Inferred to Match the Original Dataset (concurrently)	RNA-Seq	Not Explicitly Stated	FDR < 0.05 for DE genes at the gene, isoform, and AS event levels	RNA Sequencing Study	1 significant for re-experiencing; 48 significant for avoidance (23 of those unique); 5 significant for numbing; 49 significant to hyperarousal (2 of those unique)	*IFI17* (interferon-α-inducible protein 27); *AHSP* ; *ALAS* ; *HMBS*	•The top gene associated with total PCL was *IFI17* , a gene involved in innate immunity<br>•Avoidance differed the most from other dimensions with respect to DE genes and AS events<br>•Findings supported that unique biological pathways may underpin PTSD subtypes<br>•DE analyses identified 1, 48, 15, and 49 significant genes that were associated with re-experiencing, avoidance, numbing, and hyperarousal, respectively	Include
Kuan et al. [33]	324 (Current PTSD: 81; Past PTSD: 42; Never: 201)	51.78	100	Caucasian (87.65)	World Trade Center Responders (324)	United States of America	World Trade Center Terrorist Attack	SCID; PCL-17	Not Stated	Whole Blood	DNAm Age (Horvath; Hannum; PhenoAge; GrimAge); Transcriptional Age (RNAAgeCalc)	Concurrently	RNA-Seq	Not Explicitly Stated	Not Explicitly Stated	RNA Sequencing; Clock Study	Not Stated	Not Stated	•WTC responders with current PTSD showed accelerated transcriptional aging compared to WTC responders who did not have PTSD<br>•WTC responders who had PTSD in the past did not show accelerated aging, suggesting biological age may normalize after effective treatment<br>•Transcriptional age acceleration was positively associated with the avoidance dimension of PTSD<br>•GrimAge acceleration was significantly associated with PTSD diagnosis<br>•PhenoAge, Hannum, and Horvath methylation age acceleration were not reliably related to PTSD<br>•GrimAge showed significant age acceleration with PTSD diagnosis, total PCL, avoidance, and numbing dimensions	Include
Toledo et al. [37]	324 people (Current PTSD: 81; Past PTSD: 42; Never: 201)	Current: 51.57; Past: 51.36; Never: 51.36	100	Caucasian (Current: 78.6; Past: 90.0; Never: 85.2); Other (Current: 21.4; Past: 10.0; Never: 14.8)	World Trade Center Responders(324)	United States of America	World Trade Center Terrorist Attack	PCL-C	Not Stated	Blood	RNA	Can Be Inferred to Match the Original Dataset (concurrently)	RNA-Seq	Not Explicitly Stated	FDR < 0.05	RNA Sequencing Study	12 associated with the neurotrophic signaling pathway; 9 associated with the ErbB signaling pathway; 17 associated with Parkinson's disease; 19 associated with the non-alcoholic fatty liver disease pathway	*SYT1; DOCK10; SOX5; C9orf84 (SHOC1); NOS1AP; TRIM11; NDUFA1*	•Based on differential expression, the past group is more similar to the current group than it is to the never group<br>•PTSD appears to be chronic and that psychological interventions are treatments to alleviate the symptoms<br>•12 differentially expressed genes within our data were associated with the neurotrophic signaling pathway<br>•A majority of genes in the neurotrophic signaling pathway were underexpressed in PTSD patients, which could contribute to the symptoms experienced by those with PTSD<br>•9 differentially expressed genes involved in the ErbB signaling pathway<br>•There was an upregulation of the *SYT1* gene in PTSD cohorts, which interacts with Wnt pathways<br>•PTSD shares gene expression patterns with Major Depressive Disorder, Schizophrenia, Parkinson's Disease, and Alzheimer's Disease	Include
Kuan et al. (2019) [36]	39 (Case: 20; Control:19)	Case: 53.35; Control: 49.37	100	Caucasian (Case: 85; Control: 100), Other (Case: 15; Control: 0)	World Trade Center Responders (39)	United States of America	World Trade Center Terrorist Attack	SCID	Non-Smoker (100)	Whole Blood	RNA	Concurrently	RNA-Seq	Not Explicitly Stated	FDR < 0.05	RNA Sequencing Study	3 in CD4T5; 5 in CD8T; 6 monocytes; 12 in B cells, and 3 in unsorted PBMC,	*FKBP5; PI4KAP1; PF4; REST; SEPT4;*	•Monocytes showed the largest and most distinct transcriptomic changes in PTSD<br>•The upregulation in the candidate gene *PF4* was replicated<br>•*FKBP5* and *PI4KAP1* were both upregulated, but the consistent upregulation of FKBP5 across cell types potentially indicated that the effect of PTSD on *FKBP5* may not be cell-specific<br>•It is suggested that gene expression indicative of immune dysregulation is common across several immune cell populations in PTSD	Include
Kuan et al. [32]	473 (Current: 171; Past: 100; Never: 202)	Current/Case: 49.5; Past: 48.3; Never/Control: 50.0	100	Caucasian(Current/Case: 81.3; Past: 78.0; Never/Control: 81.7); Other(Current/Case: 18.7; Past: 22.0; Never/Control: 18.2)	World Trade Center Responders(473)	United States of America	World Trade Center Terrorist Attack	SCID	Current Smoker (Current: 16.4; Past: 10; Never: 4.5); Non-Smoker(Current: 83.6; Past: 90; Never: 95.5)	Whole Blood	DNA Methylation	Can Be Inferred to Match the Original Dataset (concurrently)	Illumina HumanMethylation450 BeadChip	Not Explicitly Stated	FDR < 0.05	EWAS	Not Stated	*ZDHHC11 CSMD2; FKBP; NR3C1; BDNF*	•PTSD was associated with pathways underpinned by differentially methylated genes, regulating neuron signaling, inflammation, and multiple aspects of physical health<br>•The cGMP-PKG signaling pathway has been identified as the top KEGG pathway for PTSD<br>•The second most significant pathway for PTSD was oxytocin signaling<br>•Several pathways related to immunity were identified, which supports evidence that PTSD is associated with altered inflammatory processes	Include
Miller et al. [38]	47	23.43	38.3	Caucasian (91.5); Asian (4.3); African American (2.1); Aboriginal/ Torres Strait Islander (2.1)	First-Year Australian Bachelor of Paramedic Science Students (47)	Australia	Traumatic Event	PCL-5	Current Smoker (10.6)	Saliva	DNA Methylation	Concurrently	Illumina EPIC	Not Explicitly Stated	Bonferroni corrected p<.05	Candidate Gene Study	1 associated with PTSD symptom severity; 3 in Post Traumatic Growth;	*FKBP5; NR3C1*	•DNA methylation of different *FKBP5* and *NR3C1* sites was significantly associated with PTSD symptom severity, PTG, and resilience<br>•DNA methylation in *FKBP5* site cg07485685 was a predictor of both PTSD symptom severity and resilience in opposite directions<br>•One CpG in the *FKBP5* gene, cg07485685, was significantly associated with PTSD symptom severity<br>•DNA methylation of promotor-associated region cg03906910 of *NR3C1* was significantly associated with heightened PTSD symptom severity<br>•Low PTG after trauma was significantly associated with both *FKBP5* and NR3C1 DNAm<br>•Resilience was significantly associated with DNAm of five sites of *NR3C1* and two sites of *FKBP5*	Include
Mehta et al. [40]	39	23.44	38.5	Caucasian (89.7)	First-Year Australian Bachelor of Paramedic Science Students (39)	Australia	Traumatic Event	PCL-5	Current (12.8)	Saliva	Epigenetic Age (Horvath Algorithm; GrimAge; SkinBloodAge)	Can Be Inferred to Match the Original Dataset (concurrently)	Illumina EPIC	Not Explicitly Stated	p <.05	EWAS; Clock Study	Not Stated	Not Stated	•Psychological distress reported pre-fieldwork trauma was significantly associated with epigenetic age acceleration before and after fieldwork trauma<br>•Cluster D symptoms were consistently associated with increased epigenetic age acceleration<br>•After trauma exposure, burnout and secondary traumatic stress were significantly associated with increased epigenetic aging<br>•A stronger sense of organizational membership before trauma exposure was associated with decreased epigenetic aging before and after fieldwork trauma<br>•Receiving emotional and instrumental social support after fieldwork trauma was associated with reduced epigenetic aging<br>•Epigenetic age acceleration was associated with components of compassion fatigue, such as burnout and secondary traumatic stress	Include

PTSD post-traumatic stress disorder, DSM-IV Diagnostic and Statistical Manual of Mental Disorders 4th Edition, CAPS Clinician Administered PTSD Scale, SCID Structured Clinical Interview for DSM-IV, PCL Posttraumatic Stress Disorder Checklist, DE differentially expressed, AS alternative splicing, WTC World Trade Center, PTG Post-traumatic growth, EWAS Epigenome Wise Association Study, DNAm DNA methylation
