# Supplementary material for: Comparing the molecular correlates of PTSD in first responders and the broader molecular PTSD literature: a hybrid review
Source: Front Psychol. 2026 Aug 20;17:1875411. doi: 10.3389/fpsyg.2026.1875411 (PMC13543159; doi:10.3389/fpsyg.2026.1875411)
Supplement: Supplementary file 2 [file Table_2.pdf]

Study (Year)	Objective	Study Design	Search Dates Covered	Databases Searched	Total Number of Studies	Number of Studies Included Focusing on PTSD & Epigenetics	Population Studied	Types of Study Designs Included	Measures used for PTSD	Samples	Quality Assessment
Rajabi et al. (2025)	To identify key pathways and molecular targets associated with post-traumatic stress disorder, particularly its war-related subtype	Systematic Review	2000 - January 2024	PubMed; Scopus; Web of Science	28	28	Military Personnel; Veterans; Trauma-Exposed Civilians	Longitudinal; Cohort; Case-Control; Cross-Sectional; Prospective Cohort; Observational	DSM-5 criteria	Whole Blood; Blood; Saliva; Serum; Post-Mortem Brain	Include
Pierce & Black (2023)	To systematically assess the current literature about prenatal epigenetic markers that lead to post-traumatic stress disorder susceptibility across the lifespan	Systematic Review	2002 - February 2021	PubMed; MEDLINE; Science Direct; Boston College School of Social Work Library Databases	33	33	"Participants were included whose caregivers engaged in substance use while pregnant or survived a traumatic event(s) while pregnant"	Not Stated	PCL-5	Not Stated	Include
Watkeys et al. (2018)	To systematically review the literature on NR3C1 DNA methylation in humans, to clarify the degree and direction of reported associations between NR3C1 methylation and (i) exposure to traumatic events, and (ii) various psychopathologies.	Systematic Review	Not Stated	Medline; PubMed	55	6	Adults; Interpersonal Violence Exposed Mothers; Military Personnel; Rwandan Genocide Survivors; Combat Veterans; Children	Not Stated	Semi- Structured Interview; DSM-IV criteria; CAPS; PCL-S; SCL-90; SRIP; PDS; SCID;	Blood; Saliva	Include
Wheater et al. (2020)	To provide a comprehensive summary of what is known about DNAm-MRI associative relationships across the life course, and to identify image features associated with differential DNA methylation	Systematic Review	Not Stated	Scopus; Web of Science; MEDLINE; EMBASE (via OVID)	60	7	Adults; Interpersonal-Violence Exposed Individuals; Veterans	Not Stated	Not Stated	Blood; Saliva	Include
Mehta et al. (2020)	To summarize the current DNA methylation and gene expression data with respect to three potential posttrauma responses: PTSD, PTG, and resilience	Systematic Review	2000 - Publication	MEDLINE; PubMed; PsycINFO	51	51	General Population; Veterans; Rwandan Genocide Survivors; Holocaust Survivors, Refugees; 9/11 Survivors	Cross-Sectional; Longitudinal	Single Scale; CAPS; SCID; PCL; PCL-C; m-PSS; PCL-M; PSS; CPSS; SRIP; CIDI; PPQ; MINI; IES-R; previous diagnosis of PTSD; Medical records/ proxy interviews	Blood; Saliva; Urine; Serum; Monocytes; Post-Mortem Brain Tissue	Include
Palma-Gudiel (2015)	To ascertain which methodology should be used to assess DNA methylation in the NR3C1 gene; (ii) to determine which CpG sites within the NR3C1 gene are definitely relevant for the etiology of stress-related disorders, according to published data; and (iii) to discuss the rationale driving current research on this topic in order to address future analysis of subjects suffering from stress-related disorders.	Critical Review		PubMed; PsycINFO; Web of Science	23	4	Not Clearly Stated	Not Stated	CTQ; An Index of Traumatic Events; Early Trauma Inventory; Checklist of 36 Traumatic Event Types	Peripheral Blood (leukocytes); Peripheral Blood (T lymphocytes); Peripheral Blood (PBMC); Saliva	Include

PTSD post-traumatic stress disorder, PTG post traumatic growth, MRI Magnetic Resonance ImagingDSM-5 Diagnostic and Statistical Manual of Mental Disorders-5, CAPS Clinician-Administered PTSD Scale, PCL Posttraumatic Stress Disorder Checklist, SCL Symptom Checklist, SRIP Self-Rating Inventory for PTSD, PDS Posttraumatic Diagnostic Scale, SCID Structured Clinical Interview for DSM-IV, PSS PTSD Symptom Scale, CPSS Child PTSD Symptom Scale for DSM-5, CIDI Composite International Diagnostic Interview, PPQ Perinatal Post-Traumatic Stress Disorder Questionnaire; MINI; IES-R Impact of Event Scale-Revised, CTQ Childhood Trauma Questionnaire Form
