# Supplementary material for: Comparing the molecular correlates of PTSD in first responders and the broader molecular PTSD literature: a hybrid review
Source: Front Psychol. 2026 Aug 20;17:1875411. doi: 10.3389/fpsyg.2026.1875411 (PMC13543159; doi:10.3389/fpsyg.2026.1875411)
Supplement: Supplementary file 3 [file Table_3.pdf]

Study (Year)	Objective	Study Design	Total number of Cohorts Included	Number of Cohorts Included focusing on PTSD	Names of Cohorts Included focusing on PTSD	Population Studied	Measures used for PTSD	Samples	Quality Assessment
Snijders et al. (2020)	To assess whether DNA methylation profiles are associated with the development of PTSD.	Meta-Analysis	3	3	MRS; Army STARRS; PRISMO	Military	CAPS; PCL-6; SRIP; PDL-C; DDRI; DEC	Whole Blood; Peripheral Blood	Include
Zhao et al. (2024)	To test whether new-onset PTSD diagnosis and changes in PTSD symptom severity over time were associated with changes in two metrics of epigenetic aging over two time points	Meta- Analysis	7	7	NCPTSD; TRACTS; Army STARRS; MRS; PRISMO; DNHS; AURORA	Veterans; Active Military; Detroit Residents; Trauma Exposed Adults	CAPS; CAPS-5; PCL; CIDI-SC; SRIP; PCL-C; PCL-5	Peripheral Blood	Include
Wolf et al. (2018)	To bring the strengths of the Psychiatric Genomics Consortium PTSD Epigenetics Workgroup to bear on the evaluation of the association between traumatic stress and accelerated DNA methylation age	Meta-Analysis	9	9	NCPTSD; TRACTS; MRS; Army STARRS; MIRECC-a; MIRECC-b; PRISMO; DNHS; GTP	Veterans; Active Military; Detroit Residents; Trauma Exposed Adults	CAPS-C; CAPS-L, CIDI-C; CIDI-L; SCID-C; SRIP-C; PCL-C; PCL-L;	Peripheral Blood	Include
Katrinli et al (2022)	To identify epigenetic signatures associated with PTSD symptom severity and changes in PTSS over time	Meta- Analysis	3	3	MRS; Army STARRS; PRISMO	Military Personnel	Not Stated	Whole Blood	Include
Breen et al. (2018)	To synthesize the existing data from transcriptome-wide gene expression studies in PTSD and to clarify their relevance to PTSD pathophysiology, while explicitly modeling sex- and trauma-related differences	Meta- Analysis	5	5	MRS; General Medical Community; Veterans Affairs Medical Center PTSD Outpatient Program; Acute Trauma Exposed ER	Emergency Room Trauma; Veterans; Adults with Trauma Exposure; Children	CAPS; DSM-IV Criteria; PSS items;	Isolated leukocytes; whole blood; Isolated CD14+ monocytes; Isolated mononuclear cells; combined	Include

MRS US Marine Resiliency Study, *Army STARRS* US Army Study to Assess Risk and Resilience in Servicemembers, *PRISMO* Prospective Research In Stress-related Military Operations, *NCPTSD* Longitudinal National Center for PTSD, *TRACTS* Translational Research Center for TBI and Stress Disorders, *DNHS* Detroit Neighborhood Health Study, *AURORA* Advancing Understanding of Recovery after Trauma, *MIRECC* Mid-Atlantic Mental Illness Research Education and Clinical Center PTSD Study, *GTP* Grady Trauma Project, *CAPS* Clinician-Administered PTSD Scale, *PCL* Posttraumatic Stress Disorder Checklist, *SRIP* Self-Rating Inventory for PTSD, *DDRI* Deployment Risk and Resilience Inventory, *DEC* deployment experiences checklist, *CIDI* Composite International Diagnostic Interview, *PSS* PTSD Symptom Scale
