# Supplementary material for: Comparing the molecular correlates of PTSD in first responders and the broader molecular PTSD literature: a hybrid review
Source: Front Psychol. 2026 Aug 20;17:1875411. doi: 10.3389/fpsyg.2026.1875411 (PMC13543159; doi:10.3389/fpsyg.2026.1875411)
Supplement: Supplementary file 4 [file Table_4.pdf]

Article Title/Authors	Were the criteria for inclusion in the sample clearly defined?	Were the study subjects and the setting described in detail?	Was the exposure measured in a valid and reliable way?	Were objective, standard criteria used for measurement of the condition?	Were confounding factors identified?	Were strategies to deal with confounding factors stated?	Were the outcomes measured in a valid and reliable way?	Was appropriate statistical analysis used?	Overall appraisal
An epigenome-wide DNA methylation study of PTSD and depression in World Trade Center responders **(Kuan et al., 2017)**	Yes	Yes	Yes	Yes	Yes	Yes	Yes	Yes	Include
Gene expression associated with PTSD in World Trade Center responders: an RNA sequencing study **(Kuan et al., 2017)**	Yes	Yes	Yes	Yes	Yes	Yes	Yes	Yes	Include
Genetic variant in CACNA1C is associated with PTSD in traumatized police officers **(Kryzewska et al., 2018)**	Yes	Yes	Yes	Yes	Yes	Yes	Yes	Yes	Include
Cell type-specific gene expression patterns associated with posttraumatic stress disorder in World Trade Center responders **(Kuan et al., 2019**)	Yes	Yes	Yes	Yes	Unclear	Unclear	Yes	Yes	Include
DNA methylation of NR3C1 and FKBP5 is associated with posttraumatic stress disorder, posttraumatic growth, and resilience. **(Miller et al., 2020)**	Yes	Yes	Yes	Yes	Unclear	Unclear	Yes	Yes	Include
Mapping the transcriptomics landscape of post-traumatic stress disorder symptom dimensions in World Trade Center responders **(Kuan et al., 2021)**	Yes	Yes	Yes	Yes	Yes	Yes	Yes	Yes	Include
PTSD is associated with accelerated transcriptional aging in World Trade Center responders **(Kuan et al., 2021)**	Yes	Yes	Yes	Yes	Yes	Yes	Yes	Yes	Include
Recalibrating the epigenetic clock after exposure to trauma: The role of risk and protective psychosocial factors. **(Mehta et al., 2022)**	Yes	Yes	Yes	Yes	Yes	Yes	Yes	Yes	Include
Differentially Expressed Genes and Pathways Involved in the Development of PTSD in World Trade Center Responders **(Toledo et al, 2022)**	Yes	Yes	Yes	Yes	Unclear	Unclear	Yes	Yes	Include
