# Supplementary material for: Comparing the molecular correlates of PTSD in first responders and the broader molecular PTSD literature: a hybrid review
Source: Front Psychol. 2026 Aug 20;17:1875411. doi: 10.3389/fpsyg.2026.1875411 (PMC13543159; doi:10.3389/fpsyg.2026.1875411)
Supplement: Supplementary file 5 [file Table_5.pdf]

Article Title/Authors	Is the review question clearly and explicitly stated?	Were the inclusion criteria appropriate for the review question?	Was the search strategy appropriate?	Were the sources and resources used to search for studies adequate?	Were the criteria for appraising studies appropriate?	Was critical appraisal conducted by two or more reviewers independently?	Were there methods to minimize errors in data extraction?	Were the methods used to combine studies appropriate?	Was the likelihood of publication bias assessed?	Were recommendations for policy and/or practice supported by the reported data?	Were the specific directives for new research appropriate?	Overall appraisal
Traumatic stress and accelerated DNA methylation age: A meta-analysis **(Wolf et al., 2018)**	Yes	Yes	Unclear	Unclear	Unclear	Yes	Yes	Yes	Unclear	Yes	Yes	Include
Genetic Alterations in War-Related Post-Traumatic Stress Disorder: A Systematic Review **(Rajabi et al., 2025)**	Yes	Yes	Yes	Yes	Yes	Yes	Yes	Yes	Yes	Yes	Yes	Include
Glucocorticoid receptor gene (NR3C1) DNA methylation in association with trauma, psychopathology, transcript expression, or genotypic variation: A systematic review **(Watkeys et al., 2018)**	Yes	Yes	Yes	Yes	Yes	Yes	Yes	Yes	Yes	Yes	Yes	Include
DNA methylation and brain structure and function across the life course: A systematic review **(Wheater et al., 2020)**	Yes	Yes	Yes	Yes	Yes	Unclear	No	Yes	Yes	Yes	Yes	Include
Glucocorticoid receptor gene (NR3C1) methylation processes as mediators of early adversity in stress-related disorders causality: A critical review **(Palma-Guidel et al., 2015)**	Yes	Yes	Yes	Yes	Yes	Unclear	No	Not Applicable	No	Yes	Yes	Include
Longitudinal epigenome-wide association studies of three male military cohorts reveal multiple CpG sites associated with post-traumatic stress disorder **(Snijders et al., 2020)**	Yes	Not Applicable	Not Applicable	Not Applicable	Not Applicable	Unclear	No	Yes	No	Yes	Yes	Include
PTSD and epigenetic aging: a longitudinal meta-analysis **(Zhao et al., 2024)**	Yes	Unclear	Not Applicable	Not Applicable	Not Applicable	Not Applicable	Unclear	Yes	No	Yes	Yes	Include
Epigenome-wide meta-analysis of PTSD symptom severity in three military cohorts implicates DNA methylation changes in genes involved in immune system and oxidative stress **(Katrinli et al., 2022)**	Yes	Yes	Not Applicable	Not Applicable	Not Applicable	Not Applicable	Yes	Yes	No	Yes	Yes	Include
A Systematic Review of DNA Methylation and Gene Expression Studies in Posttraumatic Stress Disorder, Posttraumatic Growth, and Resilience **(Mehta et al., 2020)**	Yes	Yes	Yes	Yes	Yes	Yes	Yes	Yes	Yes	Yes	Yes	Include
Stress and Susceptibility: A Systematic Review of Prenatal Epigenetic Risks for Developing Post-Traumatic Stress Disorder **(Pierce and Black, 2023)**	Yes	Yes	Yes	Yes	Yes	Unclear	Unclear	Yes	No	Yes	Yes	Include
PTSD Blood Transcriptome Mega-Analysis: Shared Inflammatory Pathways across Biological Sex and Modes of Trauma **(Breen et al, 2022)**	Yes	Yes	Yes	Yes	Yes	No	Yes	Yes	No	Yes	Yes	Include
